# High Temperatures Result in Smaller Nurseries which Lower Reproduction of Pollinators and Parasites in a Brood Site Pollination Mutualism

**DOI:** 10.1371/journal.pone.0115118

**Published:** 2014-12-18

**Authors:** Anusha Krishnan, Gautam Kumar Pramanik, Santosh V. Revadi, Vignesh Venkateswaran, Renee M. Borges

**Affiliations:** 1 Centre for Ecological Sciences, Indian Institute of Science, Bangalore, India; 2 Institute of Microscopy, Anatomy and Neurobiology, Johannes Gutenberg-University Medicine Mainz, Mainz, Germany; 3 Department of Plant Protection and Biology, Unit of Chemical Ecology, Swedish University of Agricultural Sciences, Alnarp, Sweden; East China Normal University, China

## Abstract

In a nursery pollination mutualism, we asked whether environmental factors affected reproduction of mutualistic pollinators, non-mutualistic parasites and seed production via seasonal changes in plant traits such as inflorescence size and within-tree reproductive phenology. We examined seasonal variation in reproduction in *Ficus racemosa* community members that utilise enclosed inflorescences called syconia as nurseries. Temperature, relative humidity and rainfall defined four seasons: winter; hot days, cold nights; summer and wet seasons. Syconium volumes were highest in winter and lowest in summer, and affected syconium contents positively across all seasons. Greater transpiration from the nurseries was possibly responsible for smaller syconia in summer. The 3–5°C increase in mean temperatures between the cooler seasons and summer reduced fig wasp reproduction and increased seed production nearly two-fold. Yet, seed and pollinator progeny production were never negatively related in any season confirming the mutualistic fig–pollinator association across seasons. Non-pollinator parasites affected seed production negatively in some seasons, but had a surprisingly positive relationship with pollinators in most seasons. While within-tree reproductive phenology did not vary across seasons, its effect on syconium inhabitants varied with season. In all seasons, within-tree reproductive asynchrony affected parasite reproduction negatively, whereas it had a positive effect on pollinator reproduction in winter and a negative effect in summer. Seasonally variable syconium volumes probably caused the differential effect of within-tree reproductive phenology on pollinator reproduction. Within-tree reproductive asynchrony itself was positively affected by intra-tree variation in syconium contents and volume, creating a unique feedback loop which varied across seasons. Therefore, nursery size affected fig wasp reproduction, seed production and within-tree reproductive phenology via the feedback cycle in this system. Climatic factors affecting plant reproductive traits cause biotic relationships between plants, mutualists and parasites to vary seasonally and must be accorded greater attention, especially in the context of climate change.

## Introduction

Abiotic factors can influence the nature and strength of mutualisms either by affecting relative densities and phenologies of mutualists and non-mutualists [1]–[3], or by altering the cost-benefit ratios between partner species by affecting nutrient availabilities [4], [5]. In plant–animal mutualisms, abiotic factors are important drivers of the biotic relationships between the interacting mutualistic partners and parasites of the mutualism [1], [5]–[8]. Seasonal environmental variations in ambient temperatures, water and light availabilities can affect plant traits such as phenology and floral size [9]–[14]. Mutualistic and parasitic organisms associated with plants would consequently experience seasonal fluctuations in resources [11], [15]–[17], which could trigger changes in patterns of biotic interactions and result in seasonal variation in the species composition of whole communities [18]. Such factors become increasingly important under the scenarios of climate change [19], [20]

In obligate brood-site pollination mutualisms such as the yucca–yucca moth or fig–fig wasp systems, the inflorescence and developing fruit also function as a nursery for the progeny of mutualistic pollinators and associated non-pollinating parasites of the mutualism [21]–[24]. For mutualists and parasites dependent upon the host plant in such systems, ovule development space, ovules or seeds are the chief reproductive resources. Therefore, seasonal variation in plant reproductive traits such as within-plant reproductive phenology or inflorescence size would lead to spatiotemporal variation in availability of brood sites for the dependent organisms. Here, we used the fig–fig wasp mutualism as a model to study the influence of climatic factors on plant reproductive traits and the reproduction of the tree and its mutualistic and parasitic fig wasp fauna. We focussed on how climatic conditions affected biotic interactions within the fig–fig wasp community through the plant traits of inflorescence size and within-tree reproductive phenology. While there is evidence for how climate change may affect mutualisms between plants and animals [25], [26], investigations on the effect of variation in climate on brood site pollination mutualisms are few [27], [28]. Also, there is a paucity of empirical investigation on how variation in climate affects tritrophic interactions [29]–[31].

The fig–fig wasp system is one with rich tritrophic interactions and is therefore ideal for studies on how changes in climate may affect species interactions within this system. The globular enclosed inflorescences of the fig called syconia contain hundreds to thousands of uniovulate flowers that develop into seeds or function as brood sites for unique assemblages of pollinating and parasitic fig wasps [23], [24], [32]. Since syconium size is a fairly good indicator of flower numbers, it is unsurprising that productivity of wasps and seeds is higher in larger syconia [33]–[35]. However, the impact of abiotic climatic factors on seasonal variations in syconium size and hence their effects on wasp and seed productivity via changes in inflorescence size have not been investigated. Furthermore, productivity of wasps and seeds is also dependent upon the availability of adult pollinator wasps, and the survival of wasp progeny under the temperature regime of their development. In tropical climates, higher ambient temperatures and lower humidity in summers are likely to heighten adult fig wasp mortalities, reduce their life spans and lower their ovipositing capabilities [27], [33], [36], [37]. Dunn et al. [36] hypothesised and Wang et al. [33], [37] demonstrated that under tropical conditions, variable adult pollinator life spans can cause seasonal variations in the ratios of seed to pollinator production. Since these ratios depend on ovule availability and extent of pollinator oviposition [38], [39], seasonal variation in adult pollinator survival times correspondingly affected the mutualism differentially—positive relationships between pollinator progeny and seed production existed in summers, whereas the opposite trend was observed in winter [37]. Although no studies have explored the effects of extrinsic climatic factors on fig wasp larval survival, such factors can affect the viability of mutualistic progeny in brood-site pollination systems [40]. Additionally, although variations in fig–pollinator relationships are affected by parasitic fig wasps that also use the fig syconium as a brood site [35], [41]–[44], the effects of climatic factors on the reproduction of such non-mutualists and their subsequent impact on the mutualism have not been explored.

Seasonality may also have an important effect on the reproductive phenology of monoecious figs (those in which female and male flower phases are temporally separated within a syconium), resulting in syconium production only when conditions are favourable. Such seasonally-dictated clustering of syconium production across trees could result in seasonal gaps in flowering during which emerging populations of short-lived adult pollinators would have no available brood sites, thereby causing local pollinator extinction [45]. Under such conditions, the seasonality hypothesis of within-tree reproductive asynchrony predicts that overlaps of male and female flower production within trees, such that syconia from which wasp progeny are emerging carrying pollen (male function of tree) coincide with syconia within which wasps can pollinate (female function of tree) and reproduce, allows trees to maintain a pollinator population [46], [47]. Alternatively, within-tree reproductive asynchrony could also extend the duration of male and female phases to increase probabilities of sexual phase overlaps between trees [45], [48], [49]. Hence, in tropical areas, higher instances of within-tree reproductive asynchrony in *Ficus* could be expected to occur in the harsher environmental conditions of summer. Non-pollinating parasitic wasps also vary in adult longevity and egg deposition strategies [50] making them more or less vulnerable to spatiotemporal variation in brood site availability. Within-tree reproductive asynchrony can cause variable spatiotemporal availabilities of oviposition resources, which could lead to differential oviposition and thereby occupancy of syconia by pollinators and parasites based on their biology. This would cause intra-crop variation in the wasp and seed composition of syconia, resulting in varied developmental times of these syconia [35], which are likely to further affect within-tree phenology. Thus, it is probable that within-tree reproductive asynchrony and wasp reproduction are involved in a unique and complex feedback cycle. Environmental conditions such as ambient temperatures can affect feedback loops in a community by affecting the reproduction of mutualists and parasites [51]. Since variable environmental conditions are likely to affect pollinator and parasitic fig wasp reproduction as well as within-plant asynchrony, it is likely that seasonality would also affect the feedback cycle in this system.

We investigated the effects of seasonal variation in climatic conditions on the reproduction of a fig community using *Ficus racemosa* and its associated fig wasp fauna as our model system. Our questions were addressed in two parts. The first part examined seasonal variation in (1) the plant traits of syconium volume and within-tree reproductive phenology; and (2) the number of pollinators, parasites and seeds produced per syconium. The second part investigated how season influenced (1) the effects of syconium volume and within-tree reproductive asynchrony on syconium inhabitants; (2) the relationship between seed and pollinator production; (3) the effect of parasites on pollinator and seed production; and (4) the feedback loop between within-tree reproductive asynchrony and fig wasp (pollinator and parasite) reproduction.

## Materials and Methods

### Species biology

The monoecious fig *Ficus racemosa* (subgenus *Sycomorus*) is found widely distributed across the Indo-Australasian region. The trees of *F. racemosa* reproduce aseasonally and may have 2–6 reproductive episodes or crops, each lasting for 2–3 months every year. *F. racemosa*, like all members of *Ficus* spp. produces reproductive structures in the form of enclosed inflorescences called syconia. Syconium development is divided into 5 phases [52], namely: A/pre-floral phase (undeveloped male and female flowers), B/female floral phase (female flowers receptive to pollination), C/interfloral phase (seeds and wasp progeny development within syconia), D/male floral phase (maturation of anthers and wasps, mating of wasps within the syconium, collection of pollen and exiting of natal syconia by female wasps through exit holes chewed by male pollinators) and E/post-floral phase (ripening of syconia and attraction of seed dispersers). The mutualistic pollinator of *F. racemosa* is the agaonid wasp *Ceratosolen fusciseps* Mayr. *F. racemosa* syconia are also parasitized by six species of non-pollinating fig wasps belonging to the subfamilies Sycophaginae and Sycoryctinae, namely, the gallers – *Apocryptophagus stratheni* Joseph, *Apocryptophagus testacea* Mayr, *Apocryptophagus fusca* Girault; and the parasitoids – *Apocryptophagus agraensis* Joseph, *Apocrypta westwoodi* Grandi and *Apocrypta* sp. 2 [50], [53], [54]. In this system, only pollinators enter the syconium to pollinate and oviposit; oviposition by all non-pollinating parasitic wasp species is from the syconium surface [55]. The various parasites oviposit into the syconia at different stages of syconial development [55] and also locate suitable syconia based on chemical cues [56].

### Study site

**Figure 1:**
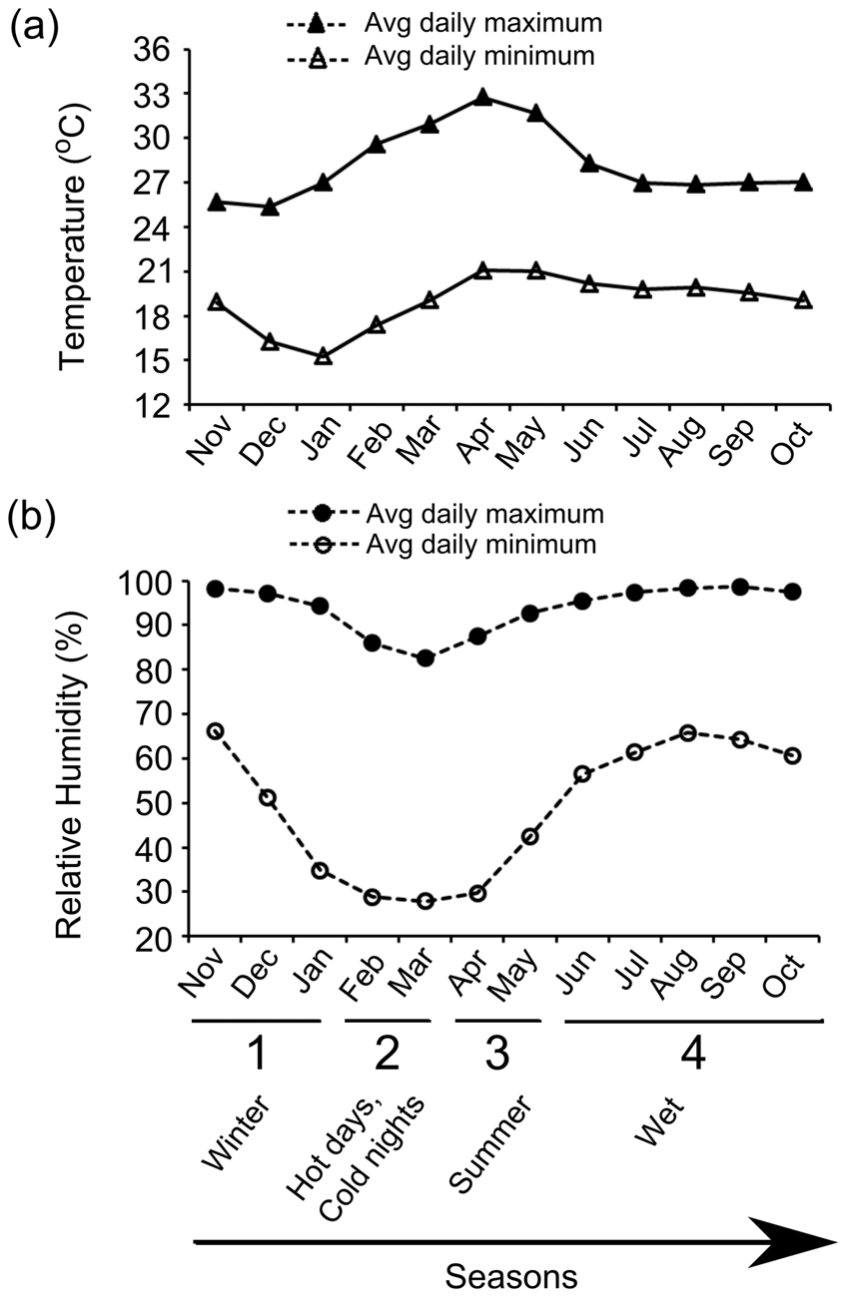
Average daily maximum and minimum values of: (a) Temperature; (b) Relative humidity for each month. Based on these values, months were grouped into 4 seasons, with 1 denoting the cold season, 2 denoting the season with hot days and cold nights, 3 denoting the hot season and 4 denoting the wet season.

The study was conducted on *F. racemosa* trees within the campus of the Indian Institute of Science (12°58′N, 77°35′E), Bangalore, India. The site is considered to have a tropical hot semi-arid climate with distinct wet and dry seasons [57]. However for this study, based on temperature and humidity data recorded over ∼2 years (between Nov 2008 to Aug 2010, obtained from the Centre for Atmospheric and Oceanic Sciences, Indian Institute of Science, Bangalore, India), we defined four seasons based on values of average daily maximum and minimum temperatures and relative humidity (RH) (Fig. 1) using PCA analysis (S1 Text, S1 Figure). These were season 1 (winter; Nov–Jan), season 2 (hot days and cold nights; Feb–Mar), season 3 (summer; Apr–May) and season 4 (wet; June–Oct). Average temperatures for each season differed from the others by at least 2°C, with the highest difference of 5°C between seasons 1 and 3 [21.5±1°C for season 1; 24.5±1°C for season 2; 26.5±1°C for season 3; and 23.5±1°C for season 4]. Season 1 or winter was defined by low temperatures; Season 2 was the driest season with the lowest humidity values; Season 3 was hot and dry with the highest temperatures and second lowest humidity values of all the seasons; Season 4 had the highest humidity values (Fig. 1). Mean temperature and RH values for each season are provided in S1 Text.

### Seasonality in within-tree reproductive phenology and syconium volume

The reproductive phenologies of 16 *Ficus racemosa* trees were observed over a period of 20 months (Nov 2008 to Aug 2010) to record within-tree patterns of flowering/fruiting. On each tree, 20 fig bunches were marked, patterns of initiation and progress of syconia through the various development phases were noted every 2–3 days over the entire observation period. A total of 94 reproductive episodes or crops were observed across this time period, with 16 in season 1, 22 in season 2, 26 in season 3 and 30 in season 4. To quantify within-tree reproductive phenology, the extent of within-tree reproductive synchrony for every reproductive episode was used. This measure was calculated using a modified form of Augspurger's index of synchrony [35], [58] where 0 indicates complete synchrony (i.e. all syconia on a tree are in the same phase) and 1 indicates complete asynchrony (syconia on a tree are equally distributed between A–E phases). A total of 1409 syconia from 16 trees across 20 months of observation were collected in the D-phase just before wasps exited. These syconia were segregated into groups corresponding to the season to which their reproductive episode was assigned. The numbers of syconia collected per season were 237 in season 1; 340 in season 2; 427 in season 3 and 405 in season 4. We obtained the ostiole–stalk insertion distance and two measures of syconium diameter (D1, D2, orthogonal to each other) using a vernier caliper. Since many syconia were ellipsoidal and not spherical, we calculated their volumes (in cm^3^) assuming them to be ellipsoids. We investigated the effects of season on within-tree asynchrony (within-tree asynchrony ∼ season) and syconium volume (volume ∼ season) in a linear mixed model (LMM) framework with tree identity as a random factor. The two dependent factors were log-transformed to achieve statistical normality. All analyses were carried out in the software R version 2.15.2 with the package *nlme*.

### Seasonality in the reproduction of fig wasps and seed production

All syconia collected for volume measurements were further used to collect information on their wasp and seed contents. Each syconium was slit and placed separately in individual capped 50 ml containers to allow wasps to exit. The syconia were then dissected to collect and count all seeds as well as exited and unexited wasps. All wasps were stored in 70% ethanol and later sorted into pollinators and parasites. For this study, we did not separate out parasites according to their species identity or biology but pooled all parasites into a single category since parasites either occupy space meant for seeds or pollinators within the nursery [43], [59]–[61]. LMMs using tree identity as a random factor and volume as a covariate were employed to investigate seasonal variation in the number of pollinators (pollinators ∼ season + volume), number of parasites (parasites ∼ season + volume) and seeds (seeds ∼ season + volume) produced per syconium. The number of pollinators per syconium was log transformed, while the number of parasites and seeds per syconium were square root transformed to achieve statistical normality.

### Seasonal variation in the relationships between plant traits, fig wasp reproduction and seed production

**Figure 2:**
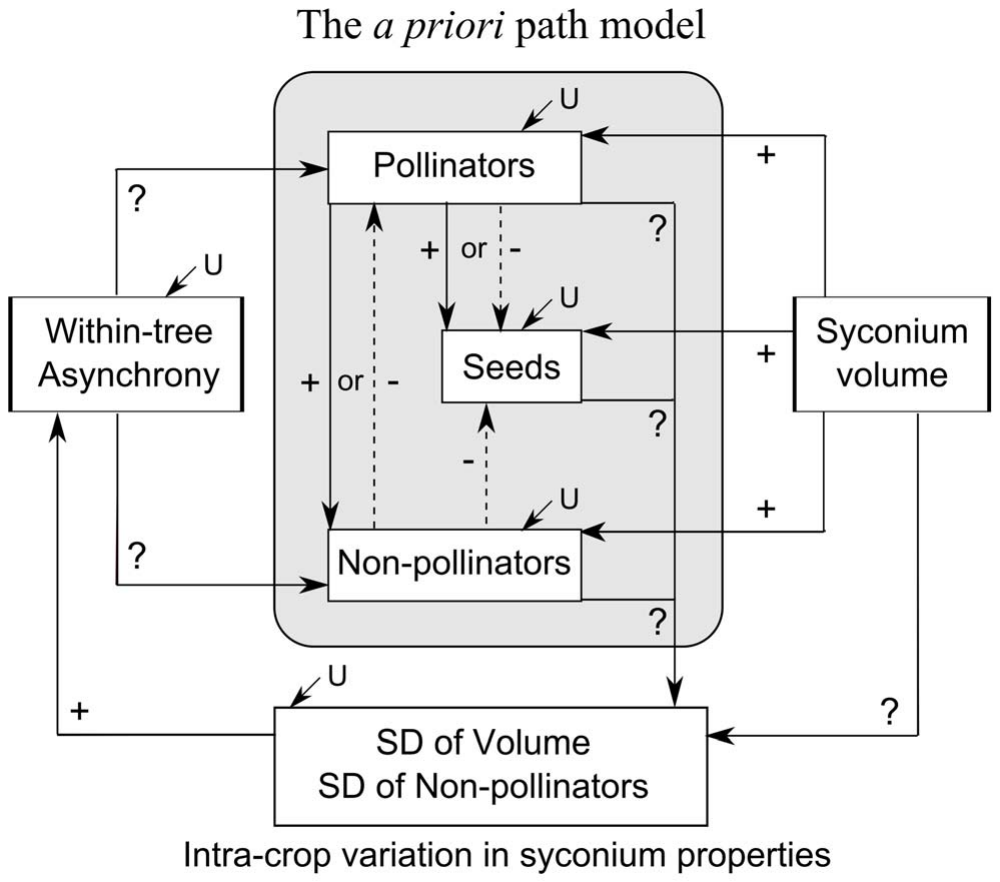
The *a priori* path model for relationships between plant reproductive traits, fig wasp and seed production. The path diagram describes the expected and unknown relationships between within-tree reproductive asynchrony, syconium volume, fig wasp and seed production. The diagram also includes the feed-back effect of variations in syconium inhabitants or volume on within-tree asynchrony. The intra-crop variations in syconium inhabitants and syconium volume were described using the standard deviation (SD) in numbers of that inhabitant and syconium volumes amongst syconia within a tree during a particular reproductive episode. The symbols ‘+’ and ‘–’ beside arrows indicate relationships expected to be positive or negative respectively, whereas ‘?’ indicates an unknown relationship (it could be positive, negative or may not exist). The magnitude of unexplained variance for a factor is indicated by ‘U’.

We used path analysis as a technique to investigate seasonal effects of within-tree reproductive asynchrony and syconium volume on wasp (pollinator and parasite) and seed production. Path analysis is a statistical tool that allows construction of complex models with multiple dependent and independent variables. This technique is based on multiple regression for the estimation of magnitude and the sign of directional relationships between variables in such models. Path analysis is particularly useful in our study system as it allows for the use of non-independent explanatory variables in data analyses. For example, in a syconium, the number of parasites acts as an explanatory variable for the number of seeds developed (Fig. 2), but, as the number of parasites itself is affected by other factors like syconium volume and within-tree asynchrony, it is a non-independent explanatory variable. Additionally, path analysis also provides for the inclusion of feed-back cycles in the data analysis for this system. Within-tree asynchrony, if it affects the inhabitants of a syconium because of its effects on wasp reproduction, could lead to variation in the development time of syconia in a tree [35]. This forms a feed-back cycle to further affect within-tree asynchrony (Fig. 2). An *a priori* path model was constructed based on the known biology of the *F. racemosa* fig wasp fauna. The rationales behind the predictions in the relationships defined in Fig. 2 are as follows:

#### (1) Effects of syconium volume and within-tree reproductive phenology on syconium inhabitants

Larger syconia with greater size and volumes have more ovules [33]–[35] which are development sites for seeds and wasps. Since syconium volume is a measure of oviposition site availability, we expected the relationships between this factor and all syconium inhabitants (pollinators, parasites and seeds) to be positive in all seasons (Fig. 2). The effect of within-tree phenology on the various syconium inhabitants could be dependent on a complex combination of various conflicting factors. Under conditions of within-tree reproductive asynchrony, individual syconia are expected to receive higher oviposition since reproductive asynchrony would lower daily availabilities of suitable oviposition sites for each type of fig wasp. Apart from this trees with strongly asynchronous fig production could also run the risk of non-pollinator populations cycling within individual trees, elevating attack rates by parasites on trees exhibiting high within-tree asynchrony [62], [63]. However, the resulting intra- or inter-specific competition for fewer oviposition sites could lower wasp progeny production [37], [64]–[66]. Furthermore, within-tree reproductive asynchrony could lower host finding efficiency and oviposition by wasps through reduced concentration of host location signals [67] which in the case of fig wasps are chemically mediated [56], [68], [69]. Although natural selection favouring high within-tree reproductive asynchrony when adult pollinators face high mortalities due to unfavourable seasonal changes has been suggested [46], [47], we could not predict the exact direction of the impact of asynchrony on wasp reproduction under the varying environmental conditions of our study (hence denoted by ‘?’ in Fig. 2).

#### (2) The relationship between pollinators and seed production

The positive or negative relationship between pollinator progeny and seed production in syconia (Fig. 2) would depend on the number of available ovules and the number of adult pollinators or foundresses entering a syconium [33], [38], [39], [70]. Under the tropical conditions of the study area, the relationship between pollinator progeny and seeds was expected to be negative at lower temperatures (such as in season 1) compared to hotter and drier situations (season 3). This is because ovipositing foundress wasp survival and hence their egg-laying capacity is expected to be higher under cooler conditions [37]. Therefore, the competition for ovules between the pollinator progeny and seeds is expected to be higher under cooler conditions.

#### (3) Effect of parasites on pollinator and seed production

Parasites were expected to have negative effects on pollinators and seeds (Fig. 2, [71]) since some parasites (gallers) compete with pollinators and seeds for ovules, while others (parasitoids) prey on pollinators. Alternatively, pollinator progeny may have a positive effect on parasites (Fig. 2) by preventing unpollinated syconia from being aborted by the tree [42] or by serving as prey for parasitoids. Adult parasitic wasps, like pollinators, could be expected to survive better in the lower temperatures and wetter conditions of tropical winters than in the hotter and drier summers [72]. Hence, syconia could be expected to experience higher parasitic wasp oviposition activity leading to a larger negative effect of parasite progeny on pollinator progeny and seed production at lower temperatures (season 1), than in hotter and drier conditions (season 3) [72].

#### (4) Feedback loop between within-tree reproductive phenology and fig wasp reproduction

Within-tree reproductive asynchrony can cause different syconia within a reproductive episode or crop to receive variable oviposition by pollinators and parasites, leading to intra-crop variation in syconium inhabitants. Variation in syconium volume, presence and number of developing parasites have been shown to affect syconium development time [35]. Therefore, intra-crop variation in syconium volume and number of parasite progeny (measured as standard deviation (SD) of volume or frequency of parasite progeny per syconium), can be expected to increase within-tree reproductive asynchrony via differential syconium development times. Hence, the feedback effect of syconium inhabitants or volume on within-tree phenology was expected to be positive (Fig. 2).

An *a priori* path model based on our predictions was tested for each season; details of the methodologies followed to obtain best-fit and most parsimonious models for each season are provided in S2 Text and S1 Table. The software LISREL 9.1 [73] was used for all path analyses. Although all variables in the data set were log transformed to improve normality, multivariate normality was not achieved. Consequently, robust maximum likelihood (RML) estimation was used to fit structural equation models to the transformed data. The relationship between any two factors was defined as a total effect partitioned into a direct effect (effect of one variable on another, represented in the model by a single causal path) and an indirect effect (a path from one variable to another which passes through some other intervening variable); these were represented as standardised path coefficients [74], [75].

## Results

### Seasonality in syconium volume and within-tree reproductive phenology

**Figure 3:**
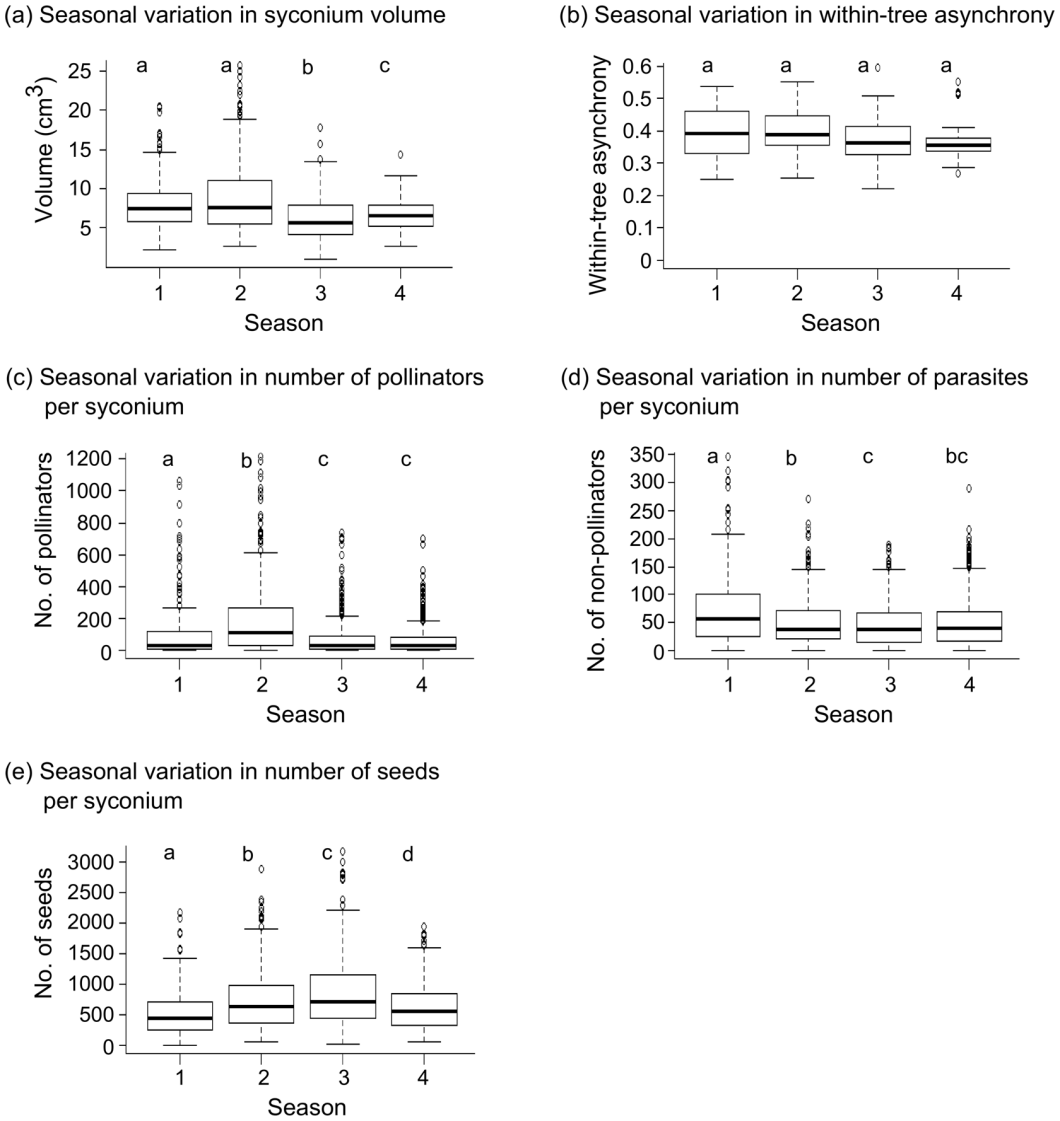
Box-plots indicating seasonal variations in plant traits, fig wasp reproduction and seed production. These are: (a) syconium volumes, (b) within-tree asynchrony values (n = 16 reproductive episodes or crops in season 1, 22 in season 2, 26 in season 3 and 30 in season 4), (c) number of pollinators per syconium, (d) number of parasites per syconium and (d) number of seeds per syconium. For measures of syconium volume, number of pollinators, parasites and seeds per syconium, n = 237 syconia in season 1, 340 in season 2, 427 in season 3 and 405 in season 4. Different letters above boxes represent significant differences at the p<0.05 level (values with the same letters were not significantly different) as according to LMM analyses using log transformed values of the first three variables and square root transformed values of the last two variables.

Syconium volumes were highest for seasons 1 (winter) and 2 (hot days and cold nights), followed by season 4 (wet), with season 3 (summer) having the smallest syconia (Fig. 3a). Heat stress appears to result in the smallest syconia. The ranges of syconium volumes were 2.3–20.5 cm^3^ (mean ± SD = 8.8±4.6) for season 1, 2.7–25.8 cm^3^ (8.1±3.3) for season 2, 1–17.9 cm^3^ (6.1±2.6) for season 3 and 2.6–14.3 cm^3^ (6.6±2) for season 4. All values, except for those between seasons 1 and 2 were significantly different from each other (Fig. 3a).

Within-tree reproductive phenology, measured as within-tree asynchrony, was similar across all four seasons (Fig. 3b). The values of within-tree asynchrony according to the modified Augspurger's index ranged between 0.25–0.54 (mean ± SD = 0.4±0.06) for season 1, 0.25–0.55 (0.4±0.08) for season 2, 0.22–0.67 (0.38±0.08) for season 3 and 0.27–0.9 (0.37±0.07) for season 4.

### Seasonality in fig wasp and seed production

The number of pollinator progeny per syconium was highest in season 2, followed by season 1 and lowest in seasons 3 and 4 (Fig. 3c). The range and mean values of pollinator progeny numbers per syconium (Fig. 3c) were: season 1 (range = 0–1059, mean ± SD = 106±182); season 2 (0–1218, 195±230); season 3 (0–739, 75±120); and season 4 (0–698, 68±100). The number of parasites produced per syconium was highest in season 1, followed by seasons 2 and 4, and lowest in season 3. The range and mean values of parasite numbers produced per syconium (Fig. 3d) were: season 1 (range = 0–347, mean ± SD = 74±67); season 2 (0–271, 52±45); season 3 (0–189, 47±40); and season 4 (0–289, 50±46). The number of seeds per syconium was highest in season 3, followed by season 2, and season 4, with the lowest values in season 1. The range and mean values of seed numbers per syconium (Fig. 3e) were: season 1 (range = 0–2176, mean ± SD = 524±359); season 2 (51–2887, 733±507); season 3 (16–3177, 868±613); and season 4 (53–1935, 611±367). Syconium volume as a covariate had a significantly positive effect on all syconium contents, i.e., on the number of pollinators (df = 1389, likelihood ratio statistic = 493.02, p<0.001), parasites (df = 1389, likelihood ratio statistic  = 123.87, p<0.001) and seeds per syconium (df = 1389, likelihood ratio statistic  =  281.08, p<0.001). Details of LMM output results are listed in S2 Table. The results of similar analyses using proportions of seeds and wasps instead of counts also showed similar patterns (S2 Figure and S3 Table). However, LMM models using proportion data were less reliable than those using count data as the proportion data models exhibited high heteroscedasticity.

### Seasonal variation in the relationships between plant traits, fig wasp reproduction and seed production

The relationships between the various factors as predicted in the *a priori* model (Fig. 2) varied with season and are summarised below.

#### (1) Effects of syconium volume and within-tree reproductive phenology on syconium inhabitants

**Figure 4:**
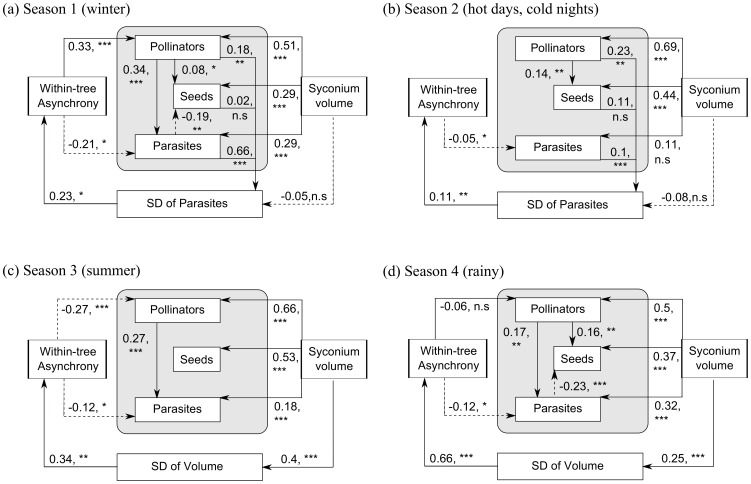
Path diagrams representative of the best-fit models for each season. These path models describe the relationships between various factors for: (a) Season 1 (winter), (b) Season 2 (hot days and cold nights), (c) Season 3 (summer) and (d) Season 4 (wet). Measures of syconium volume, number of pollinators, parasites and seeds per syconium for the path analyses were obtained from 237 syconia in season 1, 340 in season 2, 427 in season 3 and 405 in season 4. The intra-crop variations in syconium inhabitants and syconium volume were described using the standard deviation (SD) in numbers of that inhabitant and syconium volumes amongst syconia within a tree during a particular reproductive episode. All arrows indicate direct relationships between factors. Solid arrows indicate positive relationships and dotted arrows indicate negative relationships. Numbers next to these arrows indicate standardised path coefficients for direct effects. *** p<0.001, ** p<0.01 and >0.001, *p<0.05 and >0.01, n.s.

Syconium volume had a significantly positive effect on all syconium inhabitants (pollinators, parasites and seeds) in all seasons, except season 2, where it had a non-significant positive effect on parasites (Fig. 4). Furthermore, in all four seasons, syconium volume had the highest effect on number of pollinator progeny produced per syconium and the lowest effect on the number of parasite progeny (Fig. 4). Within-tree asynchrony had a variable effect on the number of pollinator progeny per syconium with season: it had a significant positive effect in season 1 (Fig. 4a), no effect in season 2 (Fig. 4b), a significant negative effect in season 3 (Fig. 4c), and a very mild negative and non-significant effect in season 4 (Fig. 4d). Within-tree asynchrony had a significant negative effect on the number of parasite progeny per syconium in all seasons (Fig. 4).

#### (2) The relationship between pollinators and seed production

The relationship between pollinator progeny and seed production per syconium was significant and positive in seasons 1, 2 and 4 (Figs. 4a, b, d). In season 3 the number of pollinator progeny per syconium did not affect seed production of the syconium (Fig. 4c).

#### (3) Effect of parasites on pollinator and seed production

The number of parasites produced per syconium was significantly and positively affected by number of pollinator progeny in seasons 1, 3 and 4 (Figs. 4a, c, d). In season 2, the number of pollinator progeny did not affect the number of parasite progeny produced per syconium (Fig. 4b). In seasons 1 and 4, number of parasite progeny had a significant negative impact on seed production (Figs. 4a and 4d). In seasons 2 and 3, the number of parasite progeny produced had no effect on the number of seeds produced per syconium (Figs. 4b, c).

#### (4) Feedback loop between within-tree reproductive phenology and fig wasp reproduction

In seasons 1 and 2, intra-crop variation in parasite progeny per syconium (represented by standard deviation or SD of parasites produced per syconium) had the maximum (S1 Table) and significantly positive effect on within-tree asynchrony (S4 Table, Figs. 4a and 4b). In these seasons, syconium volume and seed numbers per syconium had negligible effects on the SD of parasites, whereas the number of pollinator and parasite progeny per syconium had a significantly positive effect on this factor (Figs. 4a and 4b). In seasons 3 and 4, however, intra-crop variation in the volume of syconia (represented by SD of syconium volume) had the maximum effect (S1 Table) on within-tree asynchrony. The SD of syconium volume had a significantly positive effect on within-tree asynchrony (Figs. 4c and 4, S4d Table), and was itself affected significantly positively by syconium volumes (Figs. 4c and 4d).

All relationships mentioned in this section refer to the direct effects of one factor on the other, since indirect effects were generally either non-significant or of very low magnitude (S4 Table). Therefore, the total effects of one factor on another were of similar sign and magnitude as the direct effects (S4 Table). Magnitudes of unexplained variance (U) for each factor in each of the best fit models for seasons 1 to 4 are provided in S5 Table.

## Discussion

This study investigated whether reproductive plant traits such as syconium volume and within-tree asynchrony in the fig–fig wasp system, along with tree and fig wasp (pollinator and parasite) reproduction, were variable under different abiotic conditions. We used temperature, RH and rainfall as indicators of the abiotic environment to define four seasons in a year, namely: winter (season 1); hot days, cold nights (season 2); summer (season 3) and wet (season 4). Within-tree asynchrony was similar across seasons. Syconium volume, along with pollinator and parasite wasp production per syconium were highest in cooler conditions (seasons 1 and 2) and lowest in summer (season 3). Seed production per syconium showed an opposite trend, being highest in summer and lowest in winter. Syconium volume had a positive effect on all syconium contents in all seasons. Across seasons, within-tree asynchrony had a uniformly negative effect on parasite reproduction, whereas it affected pollinators differentially. Within-tree asynchrony had a positive effect on pollinator production per syconium in winter and a negative effect in summer. Pollinator progeny and seed production always had a positive relationship in all four seasons. Parasite progeny were found to affect seed production negatively, but showed a surprisingly positive relationship with pollinator progeny. The feedback cycle in this system was also variable across seasons, within-tree asynchrony being affected by intra-crop variations in parasite numbers (SD of parasites) in seasons 1 and 2, and syconium size (SD of volume) in seasons 3 and 4.

### Seasonal variations in the plant traits of syconium volume and within-tree reproductive phenology

Syconium volumes, which were used as a measure of syconium size, were highest for seasons 1 (winter) and 2 (hot days and cold nights) and lowest for season 3 (summer) (Fig. 3a). In tropical conditions, the regulation of internal temperatures in large syconia is essential for the survival of wasp progeny, and is heavily dependent on transpirational cooling through the syconium surface [76]. Season 3 was characterised by high ambient temperatures and low RH. Production of smaller-sized syconia would reduce dependence on transpirational cooling and optimize the use of water resources which may be limiting under these conditions. In seasons 1 and 2, with their lower ambient temperatures, the need for cooling to maintain internal syconium temperatures would be reduced, which probably allows the production and maintenance of larger syconium sizes.

Under abiotic conditions unfavourable for the survival of dispersing adult pollinators, within-tree reproductive asynchrony could help in maintaining pollinator populations by causing male–female sexual phase overlap within a tree [46], [47] or by lengthening the male and female phases of a tree to increase probabilities of sexual phase overlaps between trees [45], [48], [49]. In tropical areas, warmer temperatures and dry conditions during summer months (season 3) are likely to lower the life expectancy and survival of adult pollinator wasps [27], [37],which is when high within-tree asynchrony could be expected to occur. However, within-tree overlaps in male–female sexual phases in *F. racemosa* over this study period were rare [35] and our current results indicate that within-tree asynchrony values across seasons were not significantly different from each other (Fig. 3b). The negative effect of lower adult pollinator survival and dispersal abilities at higher temperatures [27] could be ameliorated by a reduced need to travel long distances to find a suitable host. It is possible that the relatively high population densities (14 *F. racemosa* trees per km^2^ in this study area; A. Krishnan, pers. obs.) and frequent reproduction (up to 7 crops per year; A. Krishnan, M. Ghara, pers. obs.) of *F. racemosa* may contribute to shorter wasp dispersal distances, which preclude the necessity of having high within-tree reproductive asynchrony during the hot and dry months (season 3) to ensure reproductive success for individual trees.

### Seasonal variation in fig wasp and seed production

Fig wasp reproduction and seed production per syconium varied significantly between seasons (Figs. 3c, d, e). In season 1, syconia contained high numbers of parasite and pollinator progeny, but produced the lowest number of seeds (Figs. 3c, d, e). The opposite trend occurred in season 3, where syconia had the highest seed production and contained the lowest numbers of wasp progeny (Figs. 3c, d, e). The increase in mean temperatures by 3–5°C in summer as compared to the cooler seasons (Seasons 1 and 2) could reduce average adult pollinator life spans and hence their oviposition abilities by one-third [37]. This increase in thermal stress could also affect the adult survival and oviposition by parasites in a similar way. Lower adult survival and oviposition by wasps in season 3 would explain the higher numbers of seeds produced per syconium in season 3, whereas higher adult survival of wasps and oviposition by wasps in season 1 shows the opposite pattern. Furthermore, the low number of pollinator and parasite progeny in season 3 could also be due to higher larval mortality in this season. Although no data are available on the effect of temperature and RH on the viability of fig wasp larvae, these two climatic factors play an important role in larval survival in the yucca–yucca moth brood site pollination mutualism [40] and may also be important in the fig system. In most plants, higher temperatures lead to lower seed production [77]–[79]; however, in our system, seed production was highest during the hot summer. Since fig wasp oviposition is most likely lower in this season [37], more ovules could be developing into seeds. In all, the differences in temperatures between these seasons reduced fig wasp reproduction nearly two-fold, while seed production per syconium was increased by roughly the same value. Syconia in season 4 (wet) produced low numbers of both wasps and seeds (Figs. 3c, 3d and 3e), which may be attributed to cloudy days and long periods of rainfall during this season. Wasps are reluctant to fly in olfactometer experiments during cloudy days (Y. Ranganathan and R M Borges, pers. obs.). Cloudiness and rainfall could interfere with wasp flight and navigation, such that fewer dispersing pollinators and parasites reach syconia to carry out pollination and oviposition. Proportions of seeds and wasps were also found to show similar patterns of variation as the count data (S2 Figure S2 and S3 Table). In general, the numbers and proportion of seeds per syconium were highest amongst all syconium inhabitants (pollinators, seeds and parasites) in all four seasons (Figs. 3c, 3d and 3e; S2 Figure and S3 Table). Although no studies have yet explored the direct effects of abiotic factors such as temperature on seed production in nursery pollination systems, our results imply that perhaps the abiotic climatic factors of temperature and RH during our study have a lower impact on seed production than they have on the production of pollinator and parasite wasp progeny.

### Seasonal effects of plant traits on the syconium contents

Syconium volume was generally found to have a positive effect on pollinator progeny and seed production (Fig. 4). Syconium volume, a proxy for size, is strongly correlated with the number of ovules contained in a syconium [35]. Since ovules are the principal syconium resources required by ovipositing pollinators and for seed production, this result was not surprising. Adult female pollinators are usually trapped within syconia when they enter them to pollinate and oviposit [23], [24], [32]. Therefore, unlike adult female parasites, which oviposit from outside the syconium, pollinators cannot move between syconia and the volume of the syconium lumen could affect a pollinator's oviposition ability. Syconium volume at D-phase has a positive correlation with lumen size in B-phase ([81]; A. Krishnan, pers. obs.; S3 Text, S3 and S4 Figures), which makes it an indicator of oviposition site availability as well as a reasonable estimator of an ovipositing female pollinator's survival, manoeuvrability and hence oviposition capability during B-phase. Therefore, it is unsurprising that syconium volume had the greatest effect on pollinator reproduction compared to all other syconium inhabitants (Fig. 4).

Within-tree asynchrony had no direct or indirect effect on seed production per syconium (Fig. 4, S4 Table) though it had a differential effect on pollinator reproduction with season (Fig. 4, S4 Table). It had a moderately positive effect in season 1, no effect in season 2, a mild negative effect in season 4 and a moderately negative effect in season 3 (Fig. 4). A tree exhibiting higher within-tree reproductive asynchrony would offer fewer host inflorescences at the right stage for oviposition per day, but for a greater number of days than a tree with a synchronous reproductive crop [62], [63]. Assuming that arriving adult pollinator numbers are constant over the duration of the B-phase on a tree, higher within-tree asynchrony would lead to fewer suitable syconia availability per day. Therefore, when within-tree reproductive asynchrony is high, higher numbers of adult pollinators are likely to enter a single syconium for pollination and oviposition. This daily effect is particularly strong for pollinators, which have adult lifespans of only 24 hours [50]. Furthermore, syconium volumes change with season (Fig. 3b). This could explain the gradual shifting of the relationship between within-tree asynchrony and pollinator production from positive to negative across seasons. Syconium volumes in season 1 are the highest (Fig. 3b), signifying that abundant resources for oviposition are available. Therefore, higher within-tree asynchrony, would probably lead to higher adult pollinator entry per syconium, which coupled with lower competition for oviposition resources owing to greater number of ovules, would lead to higher numbers of pollinator progeny per syconium. Interference competition amongst ovipositing pollinators within a syconium is expected to be highest at the low syconium volumes and hence low resource availability per syconium in season 3 [37], [39]. This is likely to lower pollinator progeny production per syconium, and explains the negative relationship between within-tree asynchrony and pollinator production per syconium in season 3.

Within-tree asynchrony had a negative effect on parasite reproduction across all seasons (Fig. 4). Although within-tree asynchrony could cause increased oviposition in individual syconia due to lower syconium availability per day, intra- and inter-specific competition between ovipositing parasites could decrease the number of progeny produced per syconium [80]. Within-tree asynchrony could also cause the mixing and dilution or ‘interference’ of volatile cues from non-specific syconium phases, which could further reduce parasite reproduction by reducing their host-syconium location efficiencies. This is often seen in other herbivorous and parasitoid species known to utilize volatiles in locating hosts [67].

### Seasonal variation in the relationship between pollinators and seed production

Apart from influencing the reproduction of mutualists individually, abiotic environmental conditions could also affect the association between the mutualists [1], [4], [82]. In our study, the relationship between seed and pollinator progeny production per syconium was positive in all seasons except season 3 (summer) (Fig. 4). The lack of a positive relationship between the two factors in this season could probably be attributed to: (1) the decreased number of pollinator eggs laid due to decreased survival of adult pollinators or foundresses in warmer and drier conditions [37]; or (2) decreased survival of pollinator wasp progeny in these conditions. Coupled with this, smaller syconium sizes (indicating lower oviposition resources within each syconium) in summer could also lead to higher interference competition between the ovipositing pollinator foundresses. With the exception of the summer season, since seed and pollinator progeny production per syconium were positively correlated most of the time, the fig–pollinator association in *F. racemosa* was largely mutualistic and therefore stable across the different environmental conditions.

### Seasonal effects of parasites on the reproduction of the mutualists

Parasites were expected to have a negative effect on pollinator and seed reproduction (Fig. 2) since some parasites (gallers) compete with pollinators and seeds for ovules, while others (parasitoids) prey on pollinators [43], [59]–[61]. Although parasites had negative effects on seed production in seasons 1 and 4, their relationships with pollinators were mostly positive (Fig. 4). Presence of pollinator progeny often protect syconia parasitized by galling parasites from aborting [42], and syconia receiving more pollinator foundresses (and hence having more pollinator progeny) could attract higher oviposition and reproduction by parasitoids [80]. These reasons could perhaps explain the surprisingly positive relationship observed between the number of pollinator and non-pollinating parasite progeny. However, if number of foundresses entering a syconium is controlled, parasites can have a negative effect on pollinator progeny numbers per syconium [83]. Parasites had negative effects on seed production only in seasons 1 and 4 (Fig. 4). The proportions of galler progeny per syconium were highest in seasons 1 and 4 (S4 Text, S5 Figure, S6 Table). Since progeny of galling parasites compete directly with seeds for ovules, this could explain why the negative effect of parasites on seed production was detected only in these two seasons.

### Seasonal variations in the cyclic feedback loop between within-tree asynchrony and syconium contents/volume

The feedback loop between within-tree asynchrony and syconium contents/volume was positive as predicted by the *a priori* model (Fig. 2). Since a syconium's parasitic wasp content and its volume can affect its development time, variation in these factors (SD of frequency of parasite progeny within a syconium and SD of volume, Figs. 2 and 4) among syconia in a reproductive episode can affect syconium development synchrony [35]. In seasons 1 and 2, where syconium volumes were large, intra-crop variation in parasites (SD of frequency of parasite progeny within a syconium, Figs. 4a and 4b) affected within-tree asynchrony (S1 Table, S4 Table). In seasons 3 and 4, intra-crop variation in syconium volume (SD of volume, Figs. 4c and 4d) was the main factor affecting within-tree asynchrony (S1 Table, S4 Table). It is possible that this variation between the seasons is attributable to the variation in syconium volume. Syconium development time is affected by both syconium volume, as well as its parasite content [35]. The lower syconium volumes in seasons 3 and 4 may have been a major limiting factor for wasp reproduction, leading to intra-crop variation in syconium volume becoming a stronger driving force in affecting syconium development time and thereby within-tree reproductive asynchrony than variation in parasite syconium content. In seasons 1 and 2, however, larger syconium volumes allowed more parasite reproduction, which may have had a more powerful effect on syconium development time than syconium volume. Hence intra-crop variation in syconium parasite content became the main factor influencing within-tree reproductive asynchrony in these two seasons.

## Conclusions

Overall, our study highlights the importance of seasonality in understanding relationships in plant–animal interaction systems. Seasonal variations in abiotic climatic factors can not only affect plant traits, but can also affect pollinator and parasitic fig wasp reproduction, seed production, and the relationships between them. Although levels of within-tree reproductive asynchrony were mostly similar across seasons, asynchrony had variable effects on pollinator reproduction which could be linked to seasonal variation in syconium volume. For the first time to our knowledge, we show that syconium productivity of parasitic non-pollinating fig wasps, as in pollinators, is higher at cooler temperatures. Despite substantial variation in pollinator and seed productivity per syconium across seasons, the relationship between them was never negative, indicating that the fig–pollinator relationship in this system is largely positive and hence mutualistic. Most of the effects seen on seed production and wasp reproduction were mediated by the effect of abiotic conditions on syconium size.

Fig–fig wasp systems represent tritrophic plant–herbivore–parasitoid communities since syconia support the reproduction of herbivorous mutualistic pollinators and non-mutualistic gallers along with their parasitoids [23], [24], [32]. Changes in the abundance of a species in response to changing climatic conditions are strongly dependent upon its biotic associations and trophic interactions with other organisms [29]–[31]. In this context, we see that higher temperatures have powerful effects on pollinator and parasite reproduction as well as seed production through their effects on inflorescence size and plant reproductive phenology. The higher temperatures in summer reduced fig wasp reproduction and increased seed production per syconium nearly two-fold as compared to the cooler seasons (seasons 1 and 2). Given the impending scenarios of climate change, our results are especially relevant as they show that stressful seasons can affect plant traits which in turn can affect reproduction of host plants and the mutualists and parasites obligately dependent on such plants.

## Supporting Information

S1 Figure **MDS plot obtained from PCA analysis.** MDS plot of the 94 reproductive episodes of *F. racemosa* trees, each of which was defined by environmental variables consisting of temperature and RH values across the duration of that reproductive episode. The different seasons in which these episodes occurred were season 1 (closed squares, ▪), season 2 (open circles, ○), season 3 (open squares, □) and season 4 (closed circles, •). The ellipses define the 95% confidence interval limit for each group around a barycentre calculated from the various points within that group.
(TIFF)

S2 Figure **Box-plots indicating seasonal variations in proportions of syconium inhabitants.** (a) Proportions of pollinators per syconium, (b) Proportions of parasites per syconium, (c) Proportions of seeds per syconium. Different letters above boxes represent significant differences at the p<0.05 level (values with the same letters were not significantly different) as according to LMM analyses using arc-sine transformed values. Proportion of pollinators  =  No. of pollinators/No. of (seeds + pollinators + parasites) Proportion of parasites  =  No. of parasites/No. of (seeds + pollinators + parasites) Proportion of seeds  =  No. of seeds/No. of (seeds + pollinators + parasites)
(TIFF)

S3 Figure **Measurements for external syconium volume and lumen volume.** (i) uncut syconium, where EOS  =  External Ostiole–Stalk length, ED  =  External Diameter; (ii) syconium cut to expose lumen, where IOS  =  Internal Ostiole–Stalk length, ID  =  Internal Diameter.
(TIFF)

S4 Figure **Positive correlation between external syconium volume and lumen volume.** Pearson correlation coefficient = 0.89, (t = 10.46, df = 28, p<0.0001).
(TIFF)

S5 Figure **Proportions of gallers per syconium across the different seasons.** The different letters above boxes represent significant differences at the p<0.05 level (values with the same letters were not significantly different) as according to binomial GLMM analyses using tree identity as a random factor.
(TIFF)

S1 Table **Descriptions of** ***a priori*** **and alternative path models derived for each season to obtain best-fit and most parsimonious models.** Models with maximum explanatory power in each of the two rounds of model testing for each season are highlighted in bold text.
(DOC)

S2 Table **LMM output for the analysis exploring the effect of season on within-tree reproductive asynchrony, syconium size (volume), pollinators, parasites and seed production.** Tree identity was used as the random factor in all these analyses. Log-transformed values of within-tree reproductive asynchrony, syconium size (volume), and pollinator numbers per syconium were used to achieve normality. Square root-transformed values of number of non-pollinators per syconium and seed numbers per syconium were used to achieve normality.
(DOC)

S3 Table **Details of LMM output for the analysis exploring the effect of season on proportions of syconium inhabitants.** Tree identity was used as the random factor in all these analyses. All proportion values were transformed using arc-sine transformation to achieve normality. However, all LMM models showed high levels of heteroscedasticity. Proportion of pollinators  =  No. of pollinators/No. of (seeds + pollinators + parasites) Proportion of parasites  =  No. of parasites/No. of (seeds + pollinators + parasites) Proportion of seeds  =  No. of seeds/No. of (seeds + pollinators + parasites).
(DOC)

S4 Table **Magnitudes of direct, indirect and total effects for each relationship in the best fit and most parsimonious model for seasons 1 to 4.** Magnitudes are represented as standardised path coefficients that range between −1 and +1. N/A indicates absence of the effect in that relationship. *** p<0.001, ** p<0.01 and >0.001, *p<0.05 and >0.01, n.s. p>0.5.
(DOC)

S5 Table **Magnitudes of unexplained variance (U) for each factor in each of the best fit models for seasons 1 to 4.** Magnitudes are represented as standardised path coefficients that range between −1 and +1. *** p<0.001, ** p<0.01 and >0.001, *p<0.05 and >0.01, n.s. p>0.5.
(DOC)

S6 Table **Details of binomial GLMM analysis to examine effect of season on the proportions of non-pollinating parasitic gallers per syconium.** Tree identity was used as a random factor in this analysis. The generalised linear mixed model (GLMM) was carried out using a logit link function (a binomial GLMM) with the data.
(DOC)

S1 Text **Defining seasons based on environmental conditions.**
(DOC)

S2 Text **Details of path model construction and selection.**
(DOC)

S3 Text **Relationship between external syconium volume and volume of lumen in B-phase syconia.**
(DOC)

S4 Text **Variation in proportions of non-pollinating parasitic gallers per syconium across the four seasons.**
(DOC)

## References

[1] Bentley BL (1976) Plants bearing extrafloral nectaries and the associated ant community: interhabitat differences in the reduction of herbivore damage. Ecology 57:815–820.

[2] Hegland SJ, Nielsen A, Lázaro A, Bjerknes A-L, Totland Ø (2009) How does climate warming affect plant-pollinator interactions? Ecol Lett 12:184–195. 19049509 10.1111/j.1461-0248.2008.01269.x

[3] Yang LH, Rudolf VHW (2010) Phenology, ontogeny and the effects of climate change on the timing of species interactions. Ecol Lett 13:1–10. 19930396 10.1111/j.1461-0248.2009.01402.x

[4] Johnson NC, Graham J-H, Smith FA (1997) Functioning of mycorrhizal associations along the mutualism–parasitism continuum. New Phytol 135:575–585.

[5] Kersch MF, Fonseca CR (2005) Abiotic factors and the conditional outcome of an ant–plant mutualism. Ecology 86:2117–2126.

[6] Gorden NLS, Adler LS (2013) Abiotic conditions affect floral antagonists and mutualists of *Impatiens capensis* (Balsaminaceae). Am J Bot 100:679–689. 23482480 10.3732/ajb.1200460

[7] Meindl GA, Bain DJ, Ashman TL (2013) Edaphic factors and plant–insect interactions: direct and indirect effects of serpentine soil on florivores and pollinators. Oecologia 173:1355–1366. 23839263 10.1007/s00442-013-2711-y

[8] Pringle EG, Akçay E, Raab TK, Dirzo R, Gordon DM (2013) Water stress strengthens mutualism among ants, trees, and scale insects. PLOS Biol 11:e1001705. 24223521 10.1371/journal.pbio.1001705 PMC3818173

[9] Beatley JC (1974) Phenological events and their environmental triggers in Mojave desert ecosystems. Ecology 55:856–863.

[10] Pitelka LF, Ashmun JW, Brown RL (1985) The relationships between seasonal variation in light intensity, ramet size, and sexual reproduction in natural and experimental populations of *Aster acuminatus* (Compositae). Am J Bot 72:311–319.

[11] van Schaik CP, Terborgh JW, Wright SJ (1993) The phenology of tropical forests: adaptive significance and consequences for primary consumers. Annu Rev Ecol Syst 24:353–377.

[12] Kudo G, Ida TY, Tani T (2008) Linkages between phenology, pollination, photosynthesis, and reproduction in deciduous forest understory plants. Ecology 89:321–331. 18409422 10.1890/06-2131.1

[13] Johansson J, Bolmgren K, Jonzén N (2013) Climate change and the optimal flowering time of annual plants in seasonal environments. Glob Change Biol 19:197–207. 10.1111/gcb.12006 23504731

[14] Ulian T, Mattana E, Pritchard HW, Skwierinski R (2013) Seasonality effects on plant phenology and seed ecology in *Oritrophium peruvianum* (Asteraceae), a threatened tropical Alpine species. S Afr J Bot 88:278–285.

[15] Wolda H (1978) Seasonal fluctuations in rainfall, food and abundance of tropical insects. J Anim Ecol 47:369–381.

[16] Malizia LR (2001) Seasonal fluctuations of birds, fruits, and flowers in a subtropical forest of Argentina. Condor 103:45–61.

[17] Muniz DG, Freitas AVL, Oliveira PS (2012) Phenological relationships of *Eunica bechina* (Lepidoptera: Nymphalidae) and its host plant, *Caryocar brasiliense* (Caryocaraceae), in a neotropical savanna. Stud Neotrop Fauna Environ 47:111–118.

[18] Rico-Gray V, Díaz-Castelazo C, Ramírez-Hernández A, Guimarães PR Jr, Holland JN (2012) Abiotic factors shape temporal variation in the structure of an ant–plant network. Arthropod-Plant Interact 6:289–295.

[19] Liu Y, Mu J, Niklas KJ, Li G, Sun S (2012) Global warming reduces plant reproductive output for temperate multi-inflorescence species on the Tibetan plateau. New Phytol 195:427–436. 22591333 10.1111/j.1469-8137.2012.04178.x

[20] Scaven VL, Rafferty NE (2013) Physiological effects of climate warming on flowering plants and insect pollinators and potential consequences for their interactions. Curr Zool 59:418–426. 24009624 10.1093/czoolo/59.3.418 PMC3761068

[21] Aker CL, Udovic D (1981) Oviposition and pollination behavior of the yucca moth, *Tegeticula maculata* (Lepidoptera: Prodoxidae), and its relation to the reproductive biology of *Yucca whipplei* (Agavaceae). Oecologia 49:96–101. 28309456 10.1007/BF00376905

[22] Pellmyr O, Leebens-Mack J, Huth CJ (1996) Non-mutualistic yucca moths and their evolutionary consequences. Nature 380:155–156. 8600388 10.1038/380155a0

[23] Herre EA, Jandér KC, Machado CA (2008) Evolutionary ecology of figs and their associates: Recent progress and outstanding puzzles. Annu Rev Ecol Evol Syst 39:439–458.

[24] Cook JM, Segar ST (2010) Speciation in fig wasps. Ecol Entomol 35:54–66.

[25] Warren RJ, Bradford MA (2014) Mutualism fails when climate response differs between interacting species. Glob Change Biol 20:466–74. 10.1111/gcb.12407 24399754

[26] Forrest JRK (2014) Plant–pollinator interactions and phenological change: what can we learn about climate impacts from experiments and observations? Oikos doi:10.1111/oik.01386.

[27] Jevanandam N, Goh AGR, Corlett RT (2013) Climate warming and the potential extinction of fig wasps, the obligate pollinators of figs. Biol Lett 9:10.1098/rsbl.2013.0041 PMC3645034 23515979

[28] Blatrix R, McKey D, Born C (2013) Consequences of past climate change for species engaged in obligatory interactions. CR Geosci 345:306–315.

[29] Van der Putten WH, Macel M, Visser ME (2010) Predicting species distribution and abundance responses to climate change: why it is essential to include biotic interactions across trophic levels. Phil Trans R Soc Lond B 365:2025–2034. 20513711 10.1098/rstb.2010.0037 PMC2880132

[30] Sentis A, Hemptinne JL, Brodeur J (2013) Effects of simulated heat waves on an experimental plant–herbivore–predator food chain. Glob Change Biol 19:833–842. 10.1111/gcb.12094 23504840

[31] Dyer LA, Richards LA, Short SA, Dodson CD (2013) Effects of CO2 and temperature on tritrophic interactions. PLOS ONE 8:e62528. 23638105 10.1371/journal.pone.0062528 PMC3636099

[32] Cook JM, Rasplus J-Y (2003) Mutualists with attitude: coevolving fig wasps and figs. Trends Ecol Evol 18:241–248.

[33] Wang R-W, Sun BF, Zheng Q, Shi L, Zhu L (2011) Asymmetric interaction and indeterminate fitness correlation between cooperative partners in the fig–fig wasp mutualism. J R Soc Interface 8:1487–1496. 21490005 10.1098/rsif.2011.0063 PMC3163427

[34] Suleman N, Quinnell RJ, Compton SG (2013) Variation in inflorescence size in a dioecious fig tree and its consequences for the plant and its pollinator fig wasp. Plant Syst Evol 299:927–934.

[35] Krishnan A, Borges RM (2014) Parasites exert conflicting selection pressures to affect reproductive asynchrony of their host plant in an obligate pollination mutualism. J Ecol doi:10.1111/1365-2745.12277..

[36] Dunn DW, Yu DW, Ridley J, Cook JM (2008a) Longevity, early emergence and body size in a pollinating fig wasp—implications for stability in a fig-pollinator mutualism. J Anim Ecol 77:927–935. 18624736 10.1111/j.1365-2656.2008.01416.x

[37] Wang R-W, Ridley J, Sun B-F, Zheng Q, Dunn DW, et al (2009) Interference competition and high temperatures reduce the virulence of fig wasps and stabilize a fig-wasp mutualism. PLOS ONE 4:1–11. 10.1371/journal.pone.0007802 PMC2771911 19915668

[38] Anstett M-C, Bronstein JL, Hossaert-McKey M (1996) Resource allocation: a conflict in the fig/fig wasp mutualism? J Evol Biol 9:417–428.

[39] Wang R-W, Shi L, Ai SM, Zheng Q (2008) Trade-off between reciprocal mutualists: local resource availability-oriented interaction in fig/fig wasp mutualism. J Anim Ecol 77:616–623. 18266694 10.1111/j.1365-2656.2008.01359.x

[40] Segraves KA (2003) Understanding stability in mutualisms: can extrinsic factors balance the yucca-yucca moth interaction? Ecology 84:2943–2951.

[41] Dunn DW, Segar ST, Ridley J, Chan R, Crozier RH, et al (2008) A role for parasites in stabilising the fig-pollinator mutualism. PLOS Biol 6:490–496. 10.1371/journal.pbio.0060059 PMC2265770 18336072

[42] Wang R-W, Sun B-F, Zheng Q (2010) Diffusive coevolution and mutualism maintenance mechanisms in a fig–fig wasp system. Ecology 91:1308–1316. 20503864 10.1890/09-1446.1

[43] Al-Beidh S, Dunn DW, Cook JM (2012) Spatial stratification of internally and externally non-pollinating fig wasps and their effects on pollinator and seed abundance in *Ficus burkei*. ISRN Zool doi:10.5402/2012/908560.

[44] Al-Beidh S, Dunn DW, Power SA, Cook JM (2012) Parasites and mutualism function: measuring enemy-free space in a fig–pollinator symbiosis. Oikos 121:1833–1839.

[45] Bronstein JL (1989) A mutualism at the edge of its range. Experientia 45:622–637.

[46] Ramírez BW (1970) Host specificity of fig wasps (Agaonidae). Evolution 24:680–691. 28564937 10.1111/j.1558-5646.1970.tb01804.x

[47] Janzen DH (1979) How to be a fig. Annu Rev Ecol Syst 10:13–51.

[48] Bronstein JL, Gouyon P-H, Gliddon C, Kjellberg F, Michaloud G (1990) The ecological consequences of flowering asynchrony in monoecious figs: a simulation study. Ecology 71:2145–2156.

[49] Gates DJ, Nason JD (2012) Flowering asynchrony and mating system effects on reproductive assurance and mutualism persistence in fragmented fig–fig wasp populations. Am J Bot 99:757–768. 22491002 10.3732/ajb.1100472

[50] Ghara M, Borges RM (2010) Comparative life-history traits in a fig wasp community: Implications for community structure. Ecol Entomol 35:139–148.

[51] Lombardero MJ, Ayres MP, Hofstetter RW, Moser JC, Lepzig KD (2003) Strong indirect interactions of *Tarsonemus* mites (Acarina: Tarsonemidae) and *Dendroctonus frontalis* (Coleoptera: Scolytidae). Oikos 102:243–252.

[52] Galil J, Eisikowitch D (1968) On the pollination ecology of *Ficus sycomorus* in East Africa. Ecology 49:259–269.

[53] Wang RW, Zheng Q (2008) Structure of a fig wasp community: temporal segregation of oviposition and larval diets. Symbiosis 45:113–116.

[54] Ghara M, Ranganathan Y, Krishnan A, Gowda V, Borges RM (2014) Divvying up an incubator: how parasitic and mutualistic fig wasps use space within their nursery microcosm. Arthropod–Plant Interact 8:191–203.

[55] Ranganathan Y, Ghara M, Borges RM (2010) Temporal associations in fig–wasp–ant interactions: Diel and phenological patterns. Entomol Exp Appl 137:50–61.

[56] Proffit M, Schatz B, Borges RM, Hossaert-Mckey M (2007) Chemical mediation and niche partitioning in non-pollinating fig-wasp communities. J Anim Ecol 76:296–303. 17302837 10.1111/j.1365-2656.2007.01213.x

[57] Gadgil S, Joshi NV (1983) Climatic clusters of the Indian region. J Climatol 3:47–63.

[58] Augspurger CK (1983) Phenology, flowering synchrony, and fruit set of six neotropical shrubs. Biotropica 15:257–267.

[59] West SA, Herre EA (1994) The ecology of the New World fig-parasitizing wasps Idarnes and implications for the evolution of the fig-pollinator mutualism. Proc R Soc Lond B 258:67–72.

[60] Kerdelhué C, Rasplus JY (1996) Non-pollinating Afrotropical fig wasps affect the fig-pollinator mutualism in *Ficus* within the subgenus Sycomorus. Oikos 75:3–14.

[61] Cardona W, Kattan G, Ulloa PC (2013) Non-pollinating fig wasps decrease pollinator and seed production in *Ficus andicola* (Moraceae). Biotropica 45:203–208.

[62] Frank SA (1989) Ecological and evolutionary dynamics of fig communities. Experientia 45:674–680.

[63] Cook JM, Power SA (1996) Effects of within-tree flowering asynchrony on the dynamics of seed and wasp production in an Australian fig species. J Biogeogr 23:487–493.

[64] Lawrence PO (1981) Interference competition and optimal host selection in the parasitic wasp, *Biosteres longicaudatus* . Ann Entomol Soc Am 74:540–544.

[65] van Alebeek FV, Rojas-Rousse D, Leveque L (1993) Interspecific competition between *Eupelmus vuilleti* and *Dinarmus basalis*, two solitary ectoparasitoids of *Bruchidae* larvae and pupae. Entomol Exp Appl 69:21–31.

[66] Harvey JA, Poelman EH, Tanaka T (2013) Intrinsic inter-and intraspecific competition in parasitoid wasps. Annu Rev Entomol 58:333–351. 23092242 10.1146/annurev-ento-120811-153622

[67] Randlkofer B, Obermaier E, Hilker M, Meiners T (2010) Vegetation complexity – the influence of plant species diversity and plant structures on plant chemical complexity and arthropods. Basic Appl Ecol 11:383–395.

[68] Grison-Pigé L, Bessière J-M, Hossaert-McKey M (2002) Specific attraction of fig-pollinating wasps: role of volatile compounds released by tropical figs. J Chem Ecol 28:283–295. 11925068 10.1023/a:1017930023741

[69] Proffit M, Chen C, Soler C, Bessière J-M, Schatz B, et al (2009) Can chemical signals, responsible for mutualistic partner encounter, promote the specific exploitation of nursery pollination mutualisms? – The case of figs and fig wasps. Entomol Exp Appl 131:46–57.

[70] Herre EA, West SA (1997) Conflict of interest in a mutualism: documenting the elusive fig wasp–seed trade–off. Proc R Soc Lond B 264:1501–1507.

[71] Kerdelhué C, Rossi JP, Rasplus J-Y (2000) Comparative community ecology studies on old world figs and fig wasps. Ecology 81:2832–2849.

[72] Wang RW, Yang JX, Yang DR (2005) Seasonal changes in the trade-off among fig-supported wasps and viable seeds in figs and their evolutionary implications. J Integr Plant Biol 47:144–152.

[73] Jöreskog KG, Sörbom D (2012) LISREL 9.1 for Windows [Computer Software]. *Scientific Software International Incorporated*. Lincolnwood, Illinois.

[74] Wright S (1934) The method of path coefficients. Ann Mathematical Stat 5:161–215.

[75] Mitchell RJ (1992) Testing evolutionary and ecological hypotheses using path analysis and structural equation modelling. Func Ecol 6:123–129.

[76] Patiño S, Herre EA, Tyree MT (1994) Physiological determinants of *Ficus* fruit temperature and implications for survival of pollinator wasp species: comparative physiology through an energy budget approach. Oecologia 100:13–20. 28307022 10.1007/BF00317125

[77] Barnabás B, Jäger K, Fehér A (2008) The effect of drought and heat stress on reproductive processes in cereals. Plant Cell Environ 31:11–38. 17971069 10.1111/j.1365-3040.2007.01727.x

[78] Young LW, Wilen RW, Bonham-Smith PC (2004) High temperature stress of Brassica napus during flowering reduces micro-and megagametophyte fertility, induces fruit abortion, and disrupts seed production. J Exp Bot 55:485–495. 14739270 10.1093/jxb/erh038

[79] Pagamas P, Nawata E (2008) Sensitive stages of fruit and seed development of chili pepper (*Capsicum annuum* L. var. Shishito) exposed to high-temperature stress. Sci Hortic 117:21–25.

[80] Suleman N, Raja S, Compton SG (2013) Parasitism of a pollinator fig wasp: mortalities are higher in figs with more pollinators, but are not related to local densities of figs. Ecol Entomol 38:478–484.

[81] Bronstein JL (1988) Predators of fig wasps. Biotropica 20:215–219.

[82] Barrett LG, Broadhurst LM, Thrall PH (2012) Geographic adaptation in plant–soil mutualisms: tests using *Acacia* spp. and Rhizobial bacteria. Func Ecol 26:457–468.

[83] Raja S, Suleman N, Quinnell RJ, Compton SG (2014). Interactions between pollinator and non-pollinator fig wasps: correlations between their numbers can be misleading. Entomol Sci doi:10.1111/ens.12100.

