# Supplementary figures and images for: High Temperatures Result in Smaller Nurseries which Lower Reproduction of Pollinators and Parasites in a Brood Site Pollination Mutualism
Source: PLoS One. 2014 Dec 18;9(12):e115118. doi: 10.1371/journal.pone.0115118 (PMC4270730; doi:10.1371/journal.pone.0115118)

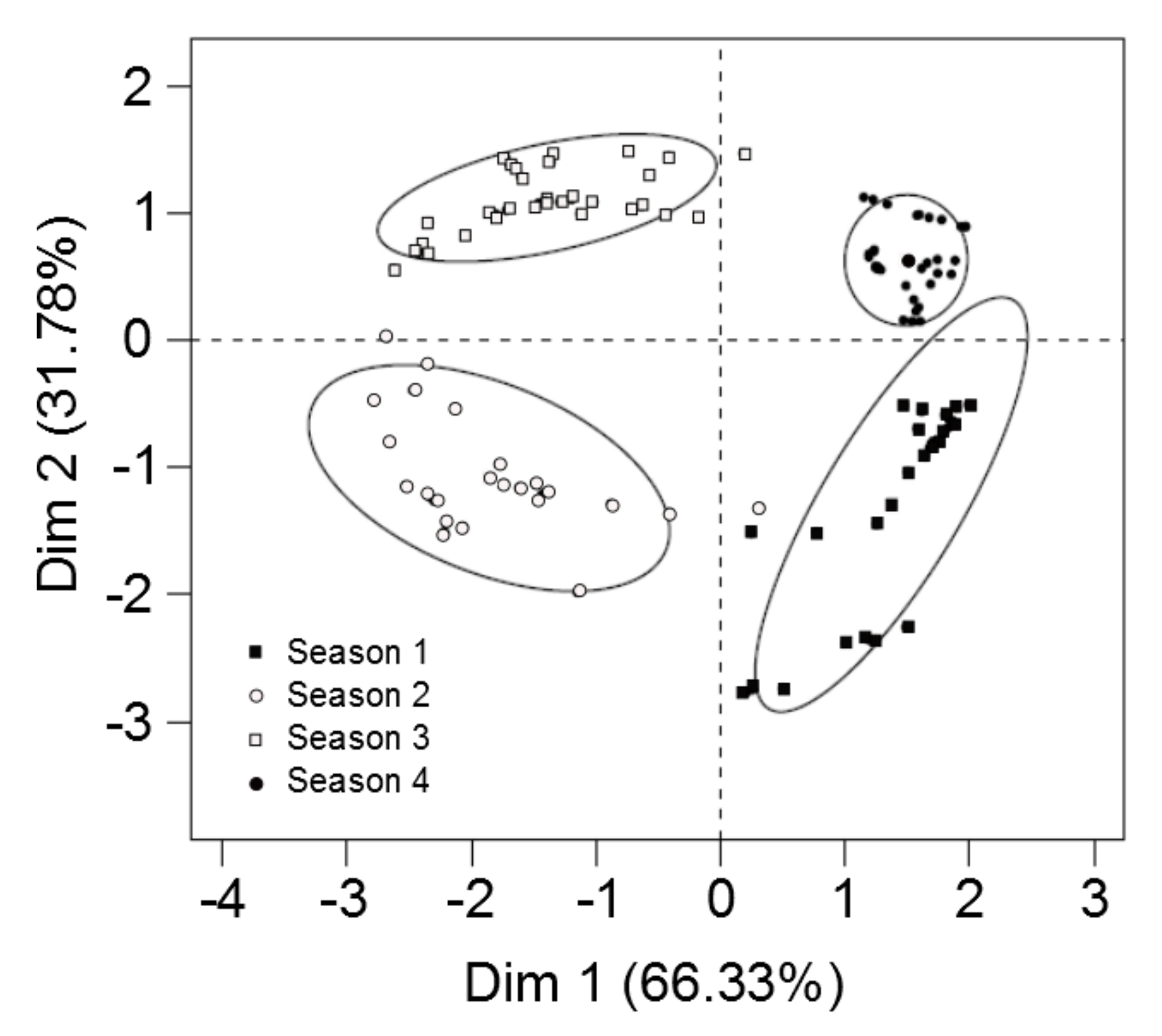

Supplement: S1 Figure — MDS plot obtained from PCA analysis. MDS plot of the 94 reproductive episodes of F. racemosa trees, each of which was defined by environmental variables consisting of temperature and RH values across the duration of that reproductive episode. The different seasons in which these episodes occurred were season 1 (closed squares, ▪), season 2 (open circles, ○), season 3 (open squares, □) and season 4 (closed circles, •). The ellipses define the 95% confidence interval limit for each group around a barycentre calculated from the various points within that group. (TIFF) [file pone.0115118.s001.tiff]

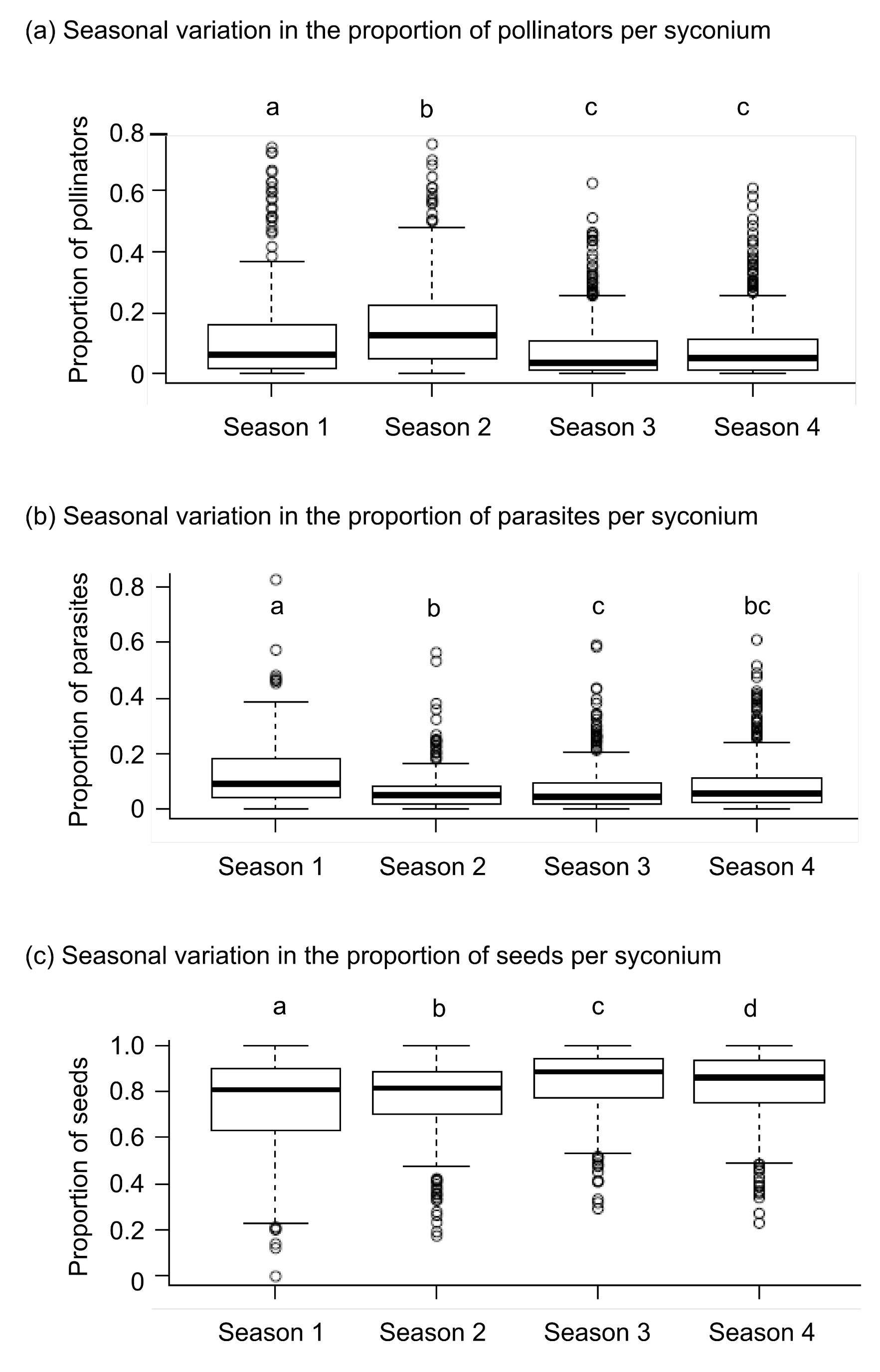

Supplement: S2 Figure — Box-plots indicating seasonal variations in proportions of syconium inhabitants. (a) Proportions of pollinators per syconium, (b) Proportions of parasites per syconium, (c) Proportions of seeds per syconium. Different letters above boxes represent significant differences at the p<0.05 level (values with the same letters were not significantly different) as according to LMM analyses using arc-sine transformed values. Proportion of pollinators = No. of pollinators/No. of (seeds + pollinators + parasites) Proportion of parasites = No. of parasites/No. of (seeds + pollinators + parasites) Proportion of seeds = No. of seeds/No. of (seeds + pollinators + parasites) (TIFF) [file pone.0115118.s002.tiff]

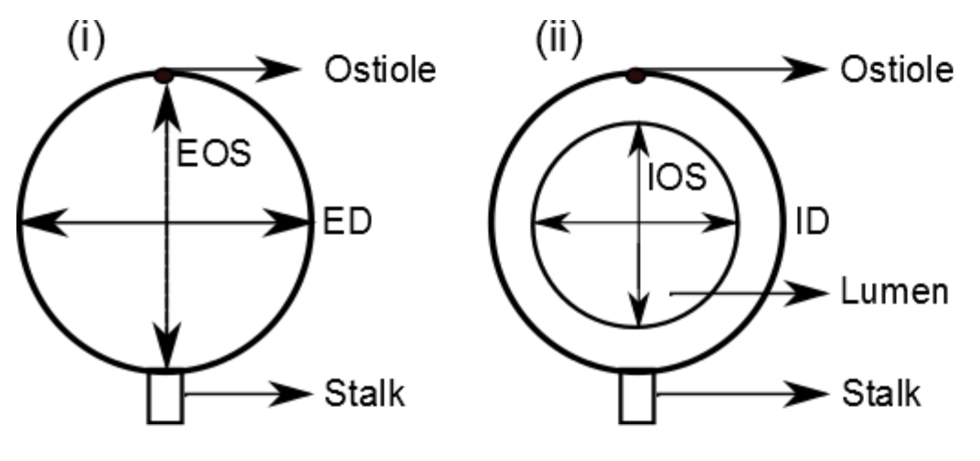

Supplement: S3 Figure — Measurements for external syconium volume and lumen volume. (i) uncut syconium, where EOS = External Ostiole–Stalk length, ED = External Diameter; (ii) syconium cut to expose lumen, where IOS = Internal Ostiole–Stalk length, ID = Internal Diameter. (TIFF) [file pone.0115118.s003.tiff]

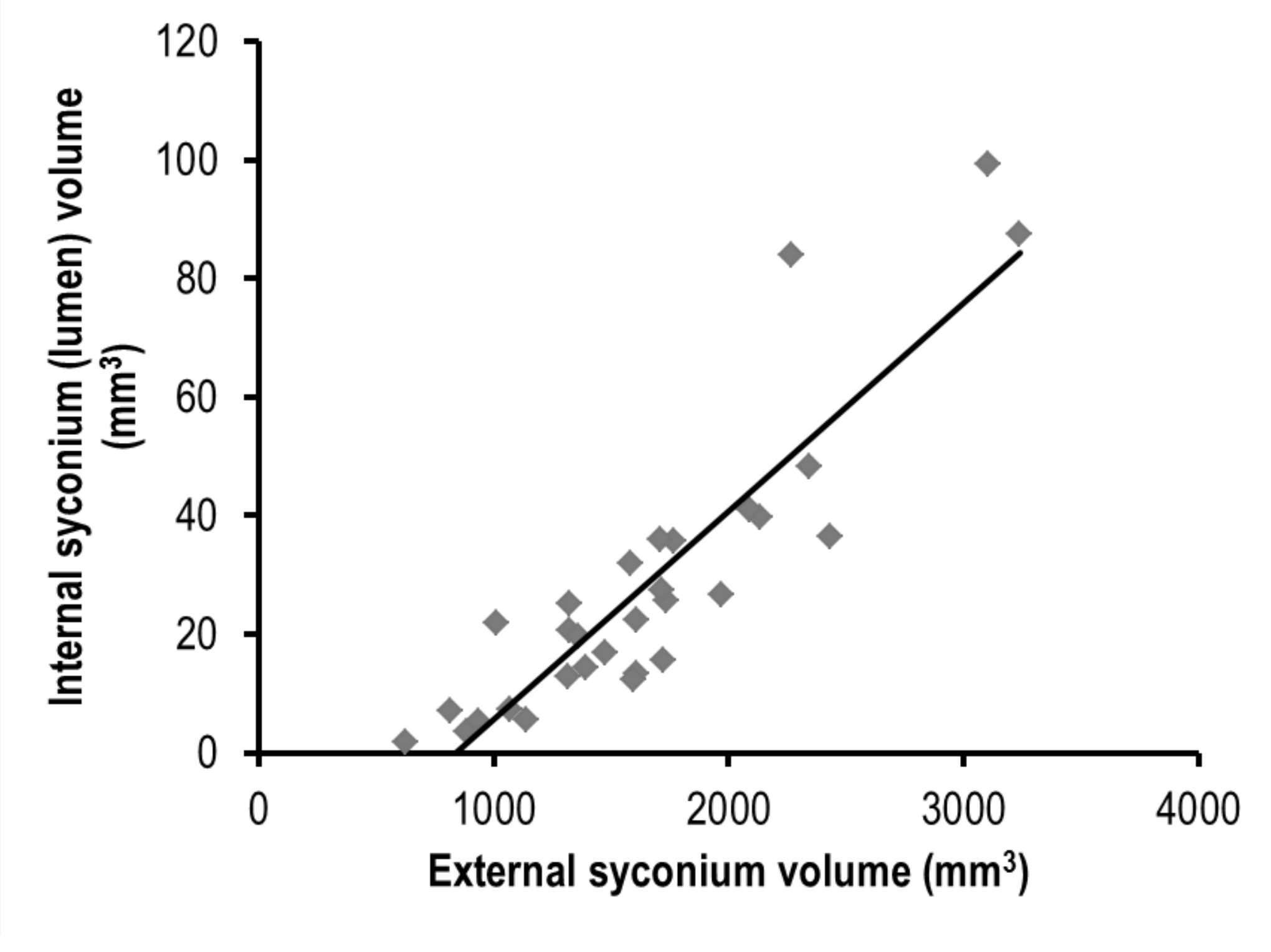

Supplement: S4 Figure — Positive correlation between external syconium volume and lumen volume. Pearson correlation coefficient = 0.89, (t = 10.46, df = 28, p<0.0001). (TIFF) [file pone.0115118.s004.tiff]

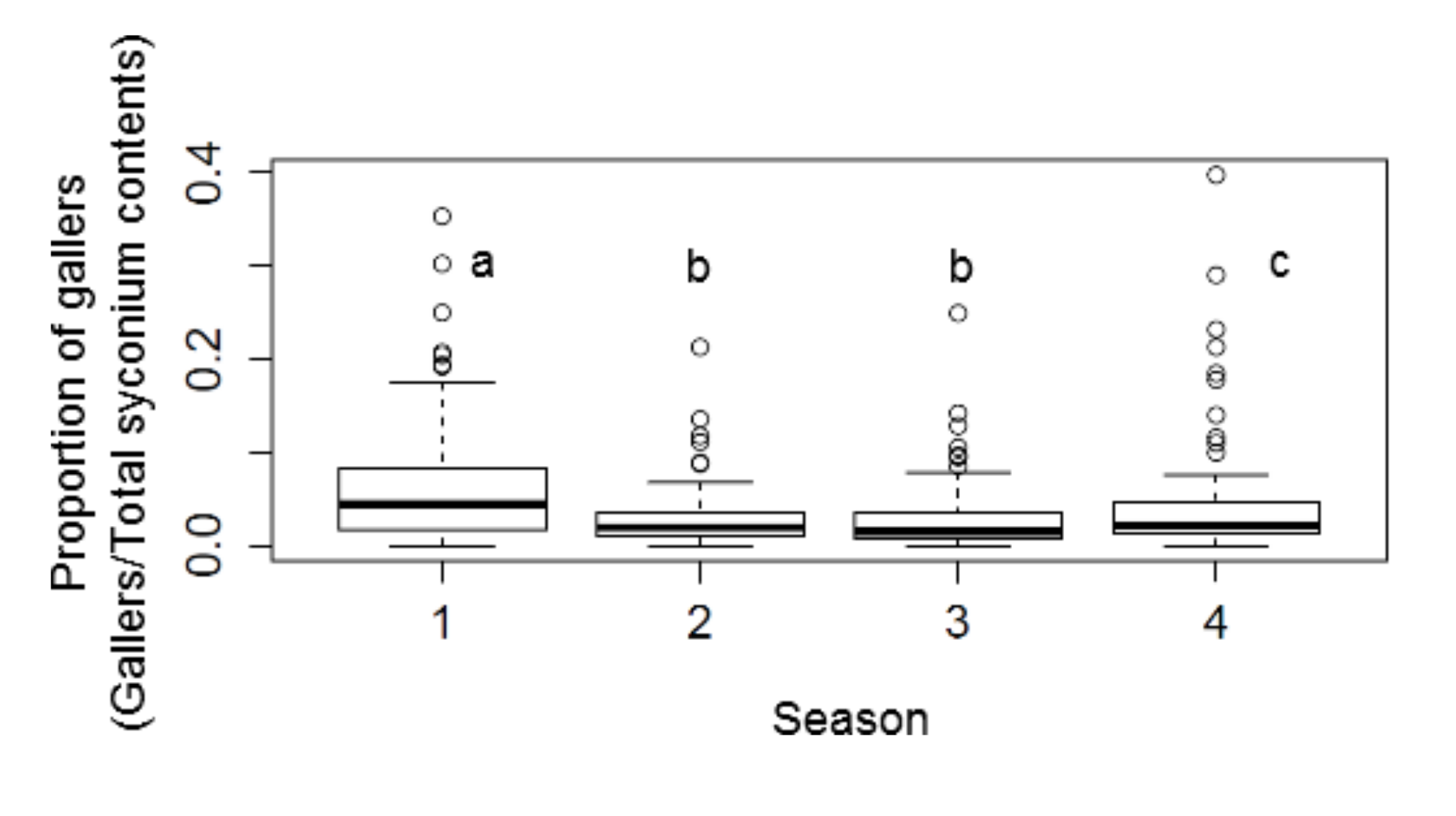

Supplement: S5 Figure — Proportions of gallers per syconium across the different seasons. The different letters above boxes represent significant differences at the p<0.05 level (values with the same letters were not significantly different) as according to binomial GLMM analyses using tree identity as a random factor. (TIFF) [file pone.0115118.s005.tiff]
