# Supplementary material for: High Temperatures Result in Smaller Nurseries which Lower Reproduction of Pollinators and Parasites in a Brood Site Pollination Mutualism
Source: PLoS One. 2014 Dec 18;9(12):e115118. doi: 10.1371/journal.pone.0115118 (PMC4270730; doi:10.1371/journal.pone.0115118)
Supplement: S1 Table — Descriptions of a priori and alternative path models derived for each season to obtain best-fit and most parsimonious models. Models with maximum explanatory power in each of the two rounds of model testing for each season are highlighted in bold text. (DOC) [file pone.0115118.s006.doc]

**Table S1. Descriptions of *a priori* and alternative path models derived for each season to obtain best-fit and most parsimonious models.** Models with maximum explanatory power in each of the two rounds of model testing for each season are highlighted in bold text.

| **Season 1 (winter)** | | | | | | | |
| --- | --- | --- | --- | --- | --- | --- | --- |
| **Model no.** | **Remove effect of** | **df** | **χ^2^-value** | **p-value** | | **RMSEA** | |
| 1 | - (a priori model) | 1 | 0.02 | 0.91 | | 0 | |
| 2 | Removing effect of within-tree asynchrony on pollinators | 2 | 48.83 | 0 | | 0.337 | |
| 3 | Removing effect of within-tree asynchrony on parasites | 2 | 1.71 | 0.43 | | 0.22 | |
| 4 | Removing effect of pollinators on parasites | 2 | 9.93 | 0.01 | | 0.188 | |
| 5 | Removing effect of parasites on seeds | 2 | 6.1 | 0.05 | | 0.107 | |
| **6** | **Removing effect of pollinators on seeds** | **2** | **0.2** | **0.9** | | **0** | |
|  | | | | | | | |
| **Model no.** | **Add effect of** | **df** | **χ^2^-value** | | **p-value** | | **RMSEA** |
| 1a | SD of volume on within-tree asynchrony | 6 | 28.8 | | 0 | | 0.123 |
| **1b** | **SD of parasites on within-tree asynchrony** | **3** | **1.6** | | **0.45** | | **0** |
|  | | | | | | | |
| **Season 2 (hot days, cold nights)** | | | | | | | |
| **Model no.** | **Remove effect of** | **df** | **χ^2^-value** | **p-value** | | **RMSEA** | |
| 1 | - (a priori model) | 1 | 5.06 | 0.02 | | 0.087 | |
| 2 | Removing effect of within-tree asynchrony on Pollinators | 2 | 6.23 | 0.04 | | 0.07 | |
| 3 | Removing effect of within-tree asynchrony on parasites | 2 | 4.56 | 0.01 | | 0.056 | |
| 4 | Removing effect of parasites on pollinators | 2 | 3.39 | 0.15 | | 0.049 | |
| 5 | Removing effect of parasites on seeds | 2 | 5.23 | 0.07 | | 0.072 | |
| 6 | Removing effect of pollinators on seeds | 2 | 6.89 | 0.02 | | 0.096 | |
| **7** | **Removing effect of within-tree asynchrony on pollinators;**  **and of parasites on pollinators and seeds** | **4** | **6.5** | **0.16** | | **0.05** | |
|  | | | | | | | |
| **Model no.** | **Add effect of** | **df** | **χ^2^-value** | | **p-value** | | **RMSEA** |
| 7a | SD of volume on within-tree asynchrony | 9 | 24.16 | | 0 | | 0.085 |
| **7b** | **SD of parasites on within-tree asynchrony** | **6** | **9.93** | | **0.13** | | **0.049** |
|  | | | | | | | |
| **Season 3 (winter)** | | | | | | | |
| **Model no.** | **Remove effect of** | **df** | **χ^2^-value** | | **p-value** | | **RMSEA** |
| 1 | - (a priori model) | 1 | 2.96 | | 0.09 | | 0.079 |
| 2 | Removing effect of within-tree asynchrony on pollinators | 2 | 53.62 | | 0 | | 0.258 |
| 3 | Removing effect of within-tree asynchrony on parasites | 2 | 9.19 | | 0.01 | | 0.096 |
| 4 | Removing effect of parasites on pollinators | 2 | 16.88 | | 0 | | 0.158 |
| 5 | Removing effect of parasites on seeds | 2 | 3.26 | | 0.2 | | 0.044 |
| 6 | Removing effect of pollinators on seeds | 2 | 3.87 | | 0.14 | | 0.061 |
| **7** | **Removing effect of parasites on seeds, and of pollinators on seeds** | **3** | **3.84** | **0.28** | | **0.041** | |
|  | | | | | | | |
| **Model no.** | **Add effect of** | **df** | **χ^2^-value** | | **p-value** | | **RMSEA** |
| **7a** | **SD of volume on within-tree asynchrony** | **7** | **5.29** | | **0.26** | | **0.039** |
| 7b | SD of parasites on within-tree asynchrony | 4 | 10.6 | | 0.16 | | 0.056 |
|  |  |  |  | |  | |  |
| **Season 4 (wet)** | | | | | | | |
| **Model no.** | **Remove effect of** | **df** | **χ^2^-value** | | **p-value** | | **RMSEA** |
| **1** | **- (a priori model)** | **1** | **0.06** | | **0.8** | | **0** |
| 2 | Removing effect of within-tree asynchrony on pollinators | 2 | 2.09 | | 0.35 | | 0.014 |
| 3 | Removing effect of within-tree asynchrony on parasites | 2 | 5.72 | | 0.06 | | 0.077 |
| 4 | Removing effect of pollinators on parasites | 2 | 8.56 | | 0.01 | | 0.104 |
| 5 | Removing effect of parasites on seeds | 2 | 19.86 | | 0 | | 0.152 |
| 6 | Removing effect of pollinators on seeds | 2 | 8.4 | | 0.01 | | 0.096 |
|  | | | | | | | |
| **Model no.** | **Add effect of** | **df** | **χ^2^-value** | | **p-value** | | **RMSEA** |
| **1a** | **SD of volume on within-tree asynchrony** | **5** | **5.86** | | **0.05** | | **0.075** |
| 1b | SD of parasites on within-tree asynchrony | 2 | 14.78 | | 0.01 | | 0.08 |
