# Supplementary material for: High Temperatures Result in Smaller Nurseries which Lower Reproduction of Pollinators and Parasites in a Brood Site Pollination Mutualism
Source: PLoS One. 2014 Dec 18;9(12):e115118. doi: 10.1371/journal.pone.0115118 (PMC4270730; doi:10.1371/journal.pone.0115118)
Supplement: S2 Table — LMM output for the analysis exploring the effect of season on within-tree reproductive asynchrony, syconium size (volume), pollinators, parasites and seed production. Tree identity was used as the random factor in all these analyses. Log-transformed values of within-tree reproductive asynchrony, syconium size (volume), and pollinator numbers per syconium were used to achieve normality. Square root-transformed values of number of non-pollinators per syconium and seed numbers per syconium were used to achieve normality. (DOC) [file pone.0115118.s007.doc]

**Table S2. LMM output for the analysis exploring the effect of season on within-tree reproductive asynchrony, syconium size (volume), pollinators, parasites and seed production.** Tree identity was used as the random factor in all these analyses. Log-transformed values of within-tree reproductive asynchrony, syconium size (volume), and pollinator numbers per syconium were used to achieve normality. Square root-transformed values of number of non-pollinators per syconium and seed numbers per syconium were used to achieve normality.

| **Within-tree asynchrony** | | | | | | | | | | | | | | | | | | | | | | | | |  |
| --- | --- | --- | --- | --- | --- | --- | --- | --- | --- | --- | --- | --- | --- | --- | --- | --- | --- | --- | --- | --- | --- | --- | --- | --- | --- |
| Linear mixed-effects model fit by maximum likelihood | | | | | | | | | | | | | | | | | | | | | | |  | |  |
| Data: basic2 | | | | | |  | | |  | | | |  | | |  | | |  | | | |  | |  |
| AIC BIC logLik | | | | | | | | | | | | |  | | |  | | |  | | | |  | |  |
| -42.73933 -27.47956 27.36966 | | | | | | | | | | | | |  | | |  | | |  | | | |  | |  |
|  | | |  | | |  | | |  | | | |  | | |  | | |  | | | |  | |  |
| Random effects: | | | | | |  | | |  | | | |  | | |  | | |  | | | |  | |  |
| Formula: ~1 | Tree | | | | | | | | |  | | | |  | | |  | | |  | | | |  | |  |
| (Intercept) Residual | | | | | | | | | | | | |  | | |  | | |  | | | |  | |  |
| StdDev: 0.1020242 0.1639254 | | | | | | | | | | | | |  | | |  | | |  | | | |  | |  |
|  | | |  | | |  | | |  | | | |  | | |  | | |  | | | |  | |  |
| Fixed effects: lE ~ season | | | | | | | | | | | | |  | | |  | | |  | | | |  | |  |
| Value Std.Error DF t-value p-value | | | | | | | | | | | | | | | | | | | | | | |  | |  |
| (Intercept) -0.9517079 0.05085004 75 -18.715971 0.0000 | | | | | | | | | | | | | | | | | | | | | | | a | |  |
| season2 0.0160697 0.05644083 75 0.284718 0.7766 | | | | | | | | | | | | | | | | | | | | | | | a | |  |
| season3 -0.0499710 0.05655243 75 -0.883622 0.3797 | | | | | | | | | | | | | | | | | | | | | | | a | |  |
| season4 -0.0530883 0.05259971 75 -1.009290 0.3161 | | | | | | | | | | | | | | | | | | | | | | | a | |  |
| Correlation: | | | | | |  | | |  | | | |  | | |  | | |  | | | |  | |  |
| (Intr) seasn2 seasn3 | | | | | | | | | | | | |  | | |  | | |  | | | |  | |  |
| season2 -0.652 | | | | | | | | | | | | |  | | |  | | |  | | | |  | |  |
| season3 -0.670 0.598 | | | | | | | | | | | | |  | | |  | | |  | | | |  | |  |
| season4 -0.697 0.631 0.628 | | | | | | | | | | | | |  | | |  | | |  | | | |  | |  |
|  | | |  | | |  | | |  | | | |  | | |  | | |  | | | |  | |  |
| Standardized Within-Group Residuals: | | | | | | | | | | | | | | | |  | | |  | | | |  | |  |
| Min Q1 Med Q3 Max | | | | | | | | | | | | | | | | | | | | | | |  | |  |
| -1.9060106 -0.5955265 -0.1105087 0.6438590 2.3308729 | | | | | | | | | | | | | | | | | | | | | | |  | |  |
|  | | |  | | |  | | |  | | | |  | | |  | | |  | | | |  | |  |
| Number of Observations: 94 | | | | | | | | | | | | |  | | |  | | |  | | | |  | |  |
| Number of Groups: 16 | | | | | | | | |  | | | |  | | |  | | |  | | | |  | |  |
|  | | | | | | | | |  | | | |  | | |  | | |  | | | |  | |  |
| **Syconium size (volume)** | | | | | | | | | | | | | | | | | | | | | | | | |  |
| Linear mixed-effects model fit by maximum likelihood | | | | | | | | | | | | | | | | | | | | | | |  | |  |
| Data: basic | | | | | |  | | | |  | | |  | | |  | | | |  | | |  | |  |
| AIC BIC logLik | | | | | | | | | | | | |  | | |  | | | |  | | |  | |  |
| 1128.115 1159.619 -558.0575 | | | | | | | | | | | | |  | | |  | | | |  | | |  | |  |
|  | |  | | | |  | | | |  | | |  | | |  | | | |  | | |  | |  |
| Random effects: | | | | | |  | | | |  | | |  | | |  | | | |  | | |  | |  |
| Formula: ~1 | Tree | | | | | | | | | |  | | |  | | |  | | | |  | | |  | |  |
| (Intercept) Residual | | | | | | | | | | | | |  | | |  | | | |  | | |  | |  |
| StdDev: 0.2389501 0.3522524 | | | | | | | | | | | | |  | | |  | | | |  | | |  | |  |
|  | |  | | |  | | |  | | | | |  | | |  | | | |  | | |  | |  |
| Fixed effects: lvol ~ season | | | | | | | | | | | | |  | | |  | | | |  | | |  | |  |
| Value Std.Error DF t-value p-value | | | | | | | | | | | | | | | | | | | | | | |  | |  |
| (Intercept) 8.956342 0.06471032 1390 138.40671 0.0000 | | | | | | | | | | | | | | | | | | | | | | | a | |  |
| season2 0.005652 0.03130096 1390 0.18058 0.8567 | | | | | | | | | | | | | | | | | | | | | | | a | |  |
| season3 -0.323269 0.03097180 1390 -10.43753 0.0000 | | | | | | | | | | | | | | | | | | | | | | | b | |  |
| season4 -0.201640 0.03007396 1390 -6.70480 0.0000 | | | | | | | | | | | | | | | | | | | | | | | c | |  |
| Correlation: | | | | |  | | |  | | | |  | | |  | | |  | | | | |  | |  |
| (Intr) seasn2 seasn3 | | | | | | | | | | | |  | | |  | | |  | | | | |  | |  |
| season2 -0.288 | | | | | | | | | | | |  | | |  | | |  | | | | |  | |  |
| season3 -0.308 0.625 | | | | | | | | | | | |  | | |  | | |  | | | | |  | |  |
| season4 -0.297 0.627 0.632 | | | | | | | | | | | |  | | |  | | |  | | | | |  | |  |
|  | |  | | |  | | |  | | | |  | | |  | | |  | | | | |  | |  |
| Standardized Within-Group Residuals: | | | | | | | | | | | | | | |  | | |  | | | | |  | |  |
| Min Q1 Med Q3 Max | | | | | | | | | | | | | | | | | | | | | | | | |  |
| -3.38464347 -0.63650442 0.01056301 0.67973012 2.88583102 | | | | | | | | | | | | | | | | | | | | | | | | |  |
|  | |  | | |  | | |  | | | |  | | |  | | |  | | |  | | | |  |
| Number of Observations: 1409 | | | | | | | | | | | |  | | |  | | |  | | |  | | | |  |
| Number of Groups: 16 | | | | | | | |  | | | |  | | |  | | |  | | |  | | | |  |
|  | | | | | | | | |  | | | |  | | |  | | |  | | | |  | |  |
| **Number of pollinators per syconium** | | | | | | | | | | | | | | | | | | | | | | | | | |
| Linear mixed-effects model fit by maximum likelihood | | | | | | | | | | | | | | | | | | | | | | | |  | |
| Data: basic | | | | |  | | |  | | | |  | | |  | | |  | | | | | |  | |
| AIC BIC logLik | | | | | | | | | | | |  | | |  | | |  | | | | | |  | |
| 2591.677 2628.431 -1288.838 | | | | | | | | | | | |  | | |  | | |  | | | | | |  | |
|  | |  | | |  | | |  | | | |  | | |  | | |  | | | | | |  | |
| Random effects: | | | | |  | | |  | | | |  | | |  | | |  | | | | | |  | |
| Formula: ~1 | Tree | | | | | | | |  | | | |  | | |  | | |  | | | | | |  | |
| (Intercept) Residual | | | | | | | | | | | |  | | |  | | |  | | | | | |  | |
| StdDev: 0.195956 0.5962696 | | | | | | | | | | | |  | | |  | | |  | | | | | |  | |
|  | |  | | |  | | |  | | | |  | | |  | | |  | | | | | |  | |
| Fixed effects: lpoll ~ season + volume | | | | | | | | | | | | | | |  | | |  | | | | | |  | |
| Value Std.Error DF t-value p-value | | | | | | | | | | | | | | | | | | | | | | | |  | |
| (Intercept) 0.2248501 0.08037392 1389 2.797551 0.0052 | | | | | | | | | | | | | | | | | | | | | | | | a | |
| season2 0.4415148 0.05280778 1389 8.360791 0.0000 | | | | | | | | | | | | | | | | | | | | | | | | b | |
| season3 0.2130876 0.05353088 1389 3.980649 0.0001 | | | | | | | | | | | | | | | | | | | | | | | | c | |
| season4 0.2241348 0.05163735 1389 4.340555 0.0000 | | | | | | | | | | | | | | | | | | | | | | | | c | |
| volume 0.0001398 0.00000576 1389 24.272362 0.0000 | | | | | | | | | | | | | | | | | | | | | | | |  | |
| Correlation: | | | | |  | | |  | | | |  | | |  | | |  | | | | | |  | |
| (Intr) seasn2 seasn3 seasn4 | | | | | | | | | | | | | | |  | | |  | | | | | |  | |
| season2 -0.362 | | | | | | | | | | | | | | |  | | |  | | | | | |  | |
| season3 -0.549 0.593 | | | | | | | | | | | | | | |  | | |  | | | | | |  | |
| season4 -0.505 0.606 0.649 | | | | | | | | | | | | | | |  | | |  | | | | | |  | |
| volume -0.601 -0.046 0.243 0.182 | | | | | | | | | | | | | | |  | | |  | | | | | |  | |
|  | |  | | |  | | |  | | | |  | | |  | | |  | | | | | |  | |
| Standardized Within-Group Residuals: | | | | | | | | | | | | | | |  | | |  | | | | | |  | |
| Min Q1 Med Q3 Max | | | | | | | | | | | | | | | | | | | | | | | |  | |
| -3.3199673 -0.6064263 0.1155948 0.7206216 2.4498283 | | | | | | | | | | | | | | | | | | | | | | | |  | |
|  | |  | | |  | | |  | | | |  | | |  | | |  | | | | | |  | |
| Number of Observations: 1409 | | | | | | | | | | | |  | | |  | | |  | | | | | |  | |
| Number of Groups: 16 | | | | | | | |  | | | |  | | |  | | |  | | | | | |  | |
|  | | | | | | | | | | | |  | | |  | | |  | | | | | |  | |
| **Number of non-pollinators per syconium** | | | | | | | | | | | | | | | | | | | | | | | | | |
| Random effects: | | | |  | | |  | | | |  | | |  | | |  | | | | |  | | | |
| Formula: ~1 | Tree | | | | | | |  | | | |  | | |  | | |  | | | | |  | | | |
| (Intercept) Residual | | | | | | | | | | |  | | |  | | |  | | | | |  | | | |
| StdDev: 1.259487 2.870946 | | | | | | | | | | |  | | |  | | |  | | | | |  | | | |
|  |  | | |  | | |  | | | |  | | |  | | |  | | | | |  | | | |
| Fixed effects: lnp ~ season + volume | | | | | | | | | | | | | |  | | |  | | | | |  | | | |
| Value Std.Error DF t-value p-value | | | | | | | | | | | | | | | | | | | | | |  | | | |
| (Intercept) 4.949369 0.4414664 1389 11.21120 0.0000 | | | | | | | | | | | | | | | | | | | | | | a | | | |
| season2 -1.061138 0.2548943 1389 -4.16305 0.0000 | | | | | | | | | | | | | | | | | | | | | | b | | | |
| season3 -0.554898 0.2592534 1389 -2.14037 0.0325 | | | | | | | | | | | | | | | | | | | | | | c | | | |
| season4 -0.721724 0.2491340 1389 -2.89693 0.0038 | | | | | | | | | | | | | | | | | | | | | | b,c | | | |
| volume 0.000318 0.0000279 1389 11.36279 0.0000 | | | | | | | | | | | | | | | | | | | | | |  | | | |
| Correlation: | | | |  | | |  | | | |  | | |  | | |  | | | | |  | | | |
| (Intr) seasn2 seasn3 seasn4 | | | | | | | | | | | | | |  | | |  | | | | |  | | | |
| season2 -0.320 | | | | | | | | | | | | | |  | | |  | | | | |  | | | |
| season3 -0.485 0.594 | | | | | | | | | | | | | |  | | |  | | | | |  | | | |
| season4 -0.446 0.607 0.648 | | | | | | | | | | | | | |  | | |  | | | | |  | | | |
| volume -0.533 -0.044 0.245 0.184 | | | | | | | | | | | | | |  | | |  | | | | |  | | | |
|  |  | | |  | | |  | | | |  | | |  | | |  | | | | |  | | | |
| Standardized Within-Group Residuals: | | | | | | | | | | | | | |  | | |  | | | | |  | | | |
| Min Q1 Med Q3 Max | | | | | | | | | | | | | | | | | | | | | | | | | |
| -3.18818978 -0.68145047 -0.01828257 0.66423183 3.31616043 | | | | | | | | | | | | | | | | | | | | | | | | | |
|  |  | | |  | | |  | | | |  | | |  | | |  | | | | |  | | | |
| Number of Observations: 1409 | | | | | | | | | | |  | | |  | | |  | | | | |  | | | |
| Number of Groups: 16 | | | | | | |  | | | |  | | |  | | |  | | | | |  | | | |
|  | | | | | | |  | | | |  | | |  | | |  | | | | |  | | | |
| **Number of seeds per syconium** | | | | | | | | | | | | | | | | | | | | | | | | | |
| Linear mixed-effects model fit by maximum likelihood | | | | | | | | | | | | | | | | | | | | |  | | | | |
| Data: basic | | | | |  | | |  | | | |  | | |  | | |  | | |  | | | | |
| AIC BIC logLik | | | | | | | | | | | |  | | |  | | |  | | |  | | | | |
| 9670.545 9707.3 -4828.273 | | | | | | | | | | | |  | | |  | | |  | | |  | | | | |
|  | |  | | |  | | |  | | | |  | | |  | | |  | | |  | | | | |
| Random effects: | | | | |  | | |  | | | |  | | |  | | |  | | |  | | | | |
| Formula: ~1 | Tree | | | | | | | |  | | | |  | | |  | | |  | | |  | | | | |
| (Intercept) Residual | | | | | | | | | | | |  | | |  | | |  | | |  | | | | |
| StdDev: 2.524362 7.3486 | | | | | | | | | | | |  | | |  | | |  | | |  | | | | |
|  | |  | | |  | | |  | | | |  | | |  | | |  | | |  | | | | |
| Fixed effects: lseed ~ season + volume | | | | | | | | | | | | | | |  | | |  | | |  | | | | |
| Value Std.Error DF t-value p-value | | | | | | | | | | | | | | | | | | | | |  | | | | |
| (Intercept) 11.432358 1.0083751 1389 11.337406 0 | | | | | | | | | | | | | | | | | | | | | a | | | | |
| season2 4.444575 0.6511136 1389 6.826113 0 | | | | | | | | | | | | | | | | | | | | | b | | | | |
| season3 6.225833 0.6604273 1389 9.426977 0 | | | | | | | | | | | | | | | | | | | | | c | | | | |
| season4 3.050398 0.6366342 1389 4.791445 0 | | | | | | | | | | | | | | | | | | | | | d | | | | |
| volume 0.001256 0.0000711 1389 17.667424 0 | | | | | | | | | | | | | | | | | | | | |  | | | | |
| Correlation: | | | | |  | | |  | | | |  | | |  | | |  | | |  | | | | |
| (Intr) seasn2 seasn3 seasn4 | | | | | | | | | | | | | | |  | | |  | | |  | | | | |
| season2 -0.356 | | | | | | | | | | | | | | |  | | |  | | |  | | | | |
| season3 -0.540 0.593 | | | | | | | | | | | | | | |  | | |  | | |  | | | | |
| season4 -0.497 0.606 0.649 | | | | | | | | | | | | | | |  | | |  | | |  | | | | |
| volume -0.592 -0.046 0.243 0.182 | | | | | | | | | | | | | | |  | | |  | | |  | | | | |
|  | |  | | |  | | |  | | | |  | | |  | | |  | | |  | | | | |
| Standardized Within-Group Residuals: | | | | | | | | | | | | | | |  | | |  | | |  | | | | |
| Min Q1 Med Q3 Max | | | | | | | | | | | | | | | | | | | | | | | | | |
| -3.55847936 -0.66603575 -0.02099008 0.65287374 2.93649728 | | | | | | | | | | | | | | | | | | | | | | | | | |
|  | |  | | |  | | |  | | | |  | | |  | | |  | | |  | | | | |
| Number of Observations: 1409 | | | | | | | | | | | |  | | |  | | |  | | |  | | | | |
| Number of Groups: 16 | | | | | | | |  | | | |  | | |  | | |  | | |  | | | | |
|  | | | | | | |  | | | |  | | |  | | |  | | | | |  | | | |
|  | | | | | | | | |  | | | |  | | |  | | |  | | | |  | | |
