# Supplementary material for: High Temperatures Result in Smaller Nurseries which Lower Reproduction of Pollinators and Parasites in a Brood Site Pollination Mutualism
Source: PLoS One. 2014 Dec 18;9(12):e115118. doi: 10.1371/journal.pone.0115118 (PMC4270730; doi:10.1371/journal.pone.0115118)
Supplement: S3 Table — Details of LMM output for the analysis exploring the effect of season on proportions of syconium inhabitants. Tree identity was used as the random factor in all these analyses. All proportion values were transformed using arc-sine transformation to achieve normality. However, all LMM models showed high levels of heteroscedasticity. Proportion of pollinators = No. of pollinators/No. of (seeds + pollinators + parasites) Proportion of parasites = No. of parasites/No. of (seeds + pollinators + parasites) Proportion of seeds = No. of seeds/No. of (seeds + pollinators + parasites). (DOC) [file pone.0115118.s008.doc]

**Table S3. LMM output for the analysis exploring the effect of season on proportions of syconium inhabitants.** Tree identity was used as the random factor in all these analyses. All proportion values were transformed using arc-sine transformation to achieve normality. However, all LMM models showed high levels of heteroscedasticity.

Proportion of pollinators = No. of pollinators/No. of (seeds + pollinators + parasites)

Proportion of parasites = No. of parasites/No. of (seeds + pollinators + parasites)

Proportion of seeds = No. of seeds/No. of (seeds + pollinators + parasites)

| **Proportion of pollinators** | | | | | | | |
| --- | --- | --- | --- | --- | --- | --- | --- |
| Linear mixed-effects model fit by maximum likelihood | | | | | | |  |
| Data: basic | |  |  |  |  |  |  |
| AIC BIC logLik | | | |  |  |  |  |
| -1798.07 -1766.567 905.0352 | | | |  |  |  |  |
|  |  |  |  |  |  |  |  |
| Random effects: | |  |  |  |  |  |  |
| Formula: ~1 | Tree | | |  |  |  |  |  |
| (Intercept) Residual | | | |  |  |  |  |
| StdDev: 0.04808026 0.1254722 | | | |  |  |  |  |
|  |  |  |  |  |  |  |  |
| Fixed effects: ppolls ~ season | | | |  |  |  |  |
| Value Std.Error DF t-value p-value | | | | | | | |
| (Intercept) 0.13198507 0.01487183 1390 8.874834 0.0000 | | | | | | | |
| season2 0.02991309 0.01111318 1390 2.691676 0.0072 | | | | | | | |
| season3 -0.06097051 0.01095690 1390 -5.564577 0.0000 | | | | | | | |
| season4 -0.03440611 0.01069094 1390 -3.218249 0.0013 | | | | | | | |
| Correlation: | |  |  |  |  |  |  |
| (Intr) seasn2 seasn3 | | | |  |  |  |  |
| season2 -0.445 | | | |  |  |  |  |
| season3 -0.473 0.624 | | | |  |  |  |  |
| season4 -0.458 0.626 0.633 | | | |  |  |  |  |
|  |  |  |  |  |  |  |  |
| Standardized Within-Group Residuals: | | | | |  |  |  |
| Min Q1 Med Q3 Max | | | | | | |  |
| -1.8584716 -0.6435095 -0.2674688 0.3079466 5.3341167 | | | | | | |  |
|  |  |  |  |  |  |  |  |
| Number of Observations: 1409 | | | |  |  |  |  |
| Number of Groups: 16 | | |  |  |  |  |  |
|  | | | | | | | |
| **Proportion of non-pollinators** | | | | | | | |
| Linear mixed-effects model fit by maximum likelihood | | | | | | |  |
| Data: basic | |  |  |  |  |  |  |
| AIC BIC logLik | | | |  |  |  |  |
| -2765.857 -2734.353 1388.928 | | | |  |  |  |  |
|  |  |  |  |  |  |  |  |
| Random effects: | |  |  |  |  |  |  |
| Formula: ~1 | Tree | | |  |  |  |  |  |
| (Intercept) Residual | | | |  |  |  |  |
| StdDev: 0.03778674 0.08890541 | | | |  |  |  |  |
|  |  |  |  |  |  |  |  |
| Fixed effects: pnps ~ season | | | |  |  |  |  |
| Value Std.Error DF t-value p-value | | | | | | | |
| (Intercept) 0.12797515 0.011309781 1390 11.315440 0e+00 | | | | | | | |
| season2 -0.05057037 0.007881046 1390 -6.416708 0e+00 | | | | | | | |
| season3 -0.04620136 0.007777187 1390 -5.940626 0e+00 | | | | | | | |
| season4 -0.03081927 0.007579235 1390 -4.066278 1e-04 | | | | | | | |
| Correlation: | |  |  |  |  |  |  |
| (Intr) seasn2 seasn3 | | | |  |  |  |  |
| season2 -0.415 | | | |  |  |  |  |
| season3 -0.442 0.624 | | | |  |  |  |  |
| season4 -0.427 0.626 0.633 | | | |  |  |  |  |
|  |  |  |  |  |  |  |  |
| Standardized Within-Group Residuals: | | | | |  |  |  |
| Min Q1 Med Q3 Max | | | | | | |  |
| -1.9444465 -0.5630555 -0.2080141 0.2787253 9.7413310 | | | | | | |  |
|  |  |  |  |  |  |  |  |
| Number of Observations: 1409 | | | |  |  |  |  |
| Number of Groups: 16 | | |  |  |  |  |  |
|  | | |  |  |  |  |  |
| **Proportion of seeds** | | | | | | | |
| Linear mixed-effects model fit by maximum likelihood | | | | | | |  |
| Data: basic | |  |  |  |  |  |  |
| AIC BIC logLik | | | |  |  |  |  |
| 78.11101 109.6148 -33.05551 | | | |  |  |  |  |
|  |  |  |  |  |  |  |  |
| Random effects: | |  |  |  |  |  |  |
| Formula: ~1 | Tree | | |  |  |  |  |  |
| (Intercept) Residual | | | |  |  |  |  |
| StdDev: 0.1047173 0.243881 | | | |  |  |  |  |
|  |  |  |  |  |  |  |  |
| Fixed effects: pseeds ~ season | | | |  |  |  |  |
| Value Std.Error DF t-value p-value | | | | | | | |
| (Intercept) 0.9099570 0.03124862 1390 29.119914 0.0000 | | | | | | | |
| season2 -0.0065286 0.02162054 1390 -0.301963 0.7627 | | | | | | | |
| season3 0.1495011 0.02133736 1390 7.006543 0.0000 | | | | | | | |
| season4 0.0822843 0.02079197 1390 3.957501 0.0001 | | | | | | | |
| Correlation: | |  |  |  |  |  |  |
| (Intr) seasn2 seasn3 | | | |  |  |  |  |
| season2 -0.412 | | | |  |  |  |  |
| season3 -0.439 0.624 | | | |  |  |  |  |
| season4 -0.424 0.626 0.633 | | | |  |  |  |  |
|  |  |  |  |  |  |  |  |
| Standardized Within-Group Residuals: | | | | |  |  |  |
| Min Q1 Med Q3 Max | | | | | | |  |
| -4.2137725 -0.6420415 0.1136443 0.7077656 2.6630983 | | | | | | |  |
|  |  |  |  |  |  |  |  |
| Number of Observations: 1409 | | | |  |  |  |  |
| Number of Groups: 16 | | |  |  |  |  |  |
|  | | |  |  |  |  |  |
|  | | | | | | | |
