# Supplementary material for: High Temperatures Result in Smaller Nurseries which Lower Reproduction of Pollinators and Parasites in a Brood Site Pollination Mutualism
Source: PLoS One. 2014 Dec 18;9(12):e115118. doi: 10.1371/journal.pone.0115118 (PMC4270730; doi:10.1371/journal.pone.0115118)
Supplement: S4 Table — Magnitudes of direct, indirect and total effects for each relationship in the best fit and most parsimonious model for seasons 1 to 4. Magnitudes are represented as standardised path coefficients that range between −1 and +1. N/A indicates absence of the effect in that relationship. *** p<0.001, ** p<0.01 and >0.001, *p<0.05 and >0.01, n.s. p>0.5. (DOC) [file pone.0115118.s009.doc]

**Table S4.** **Magnitudes of direct, indirect and total effects for each relationship in the best fit and most parsimonious model for seasons 1 to 4**.

Magnitudes are represented as standardised path coefficients that range between -1 and +1. N/A indicates absence of the effect in that relationship. *** p < 0.001, ** p < 0.01 and > 0.001, *p <0.05 and > 0.01, n.s. p > 0.5.

| **Season 1 (winter)** | | | |
| --- | --- | --- | --- |
| **Relationship between** | **Direct effects** | **Indirect effects** | **Total effects** |
| Within-tree asynchrony – Pollinators  Within-tree asynchrony – Parasites  Within-tree asynchrony – Seeds | 0.33, ***  -0.21, *  N/A | N/A  0.11, **  0.018, n.s | 0.33, ***  -0.096, n.s  0.018, n.s |
| Pollinators – Parasites  Pollinators – Seeds | 0.34, **  0.08, * | -0.009, n.s  -0.063, n.s | 0.329, **  0.03, * |
| Non-pollinators – Seeds | -0.19, ** | 0.003, n.s | -0.188, * |
| SD of parasites – Within-tree asynchrony | 0.23, * | N/A | 0.23, * |
| Volume – Pollinators  Volume – Parasites  Volume – Seeds | 0.51, ***  0.29, ***  0.29, *** | 0.03, n.s  0.17, n.s  -0.09, * | 0.54, ***  0.46, ***  0.21, ** |
| Parasites – SD of parasites  Pollinators – SD of parasites  Seeds – SD of parasites  Volume – SD of parasites | 0.66, ***  0.18, **  0.02, n.s  -0.05, n.s | N/A  N/A  N/A  0.41, *** | 0.66, ***  0.18, **  0.02, n.s  0.36, *** |
|  |  |  |  |
| **Season 2 (hot days, cold nights)** | | | |
| **Relationship between** | **Direct effects** | **Indirect effects** | **Total effects** |
| Within-tree asynchrony – Pollinators  Within-tree asynchrony – Parasites  Within-tree asynchrony – Seeds | N/A  -0.05, *  N/A | N/A  N/A  N/A | N/A  -0.05, *  N/A |
| Pollinators – Parasites  Pollinators – Seeds | N/A  0.14, ** | N/A  N/A | N/A  0.14, ** |
| Parasites – Seeds | N/A | N/A | N/A |
| SD of parasites – Within-tree asynchrony | 0.11, * | N/A | 0.11, * |
| Volume – Pollinators  Volume – Parasites  Volume – Seeds | 0.69, ***  0.11, *  0.44, *** | N/A  N/A  0.1, * | 0.69, ***  0.11, *  0.53, *** |
| Parasites – SD of parasites  Pollinators – SD of parasites  Seeds – SD of parasites  Volume – SD of parasites | 0.1, *  0.23, **  0.11, n.s -0.08, n.s | N/A  N/A  N/A  0.23, ** | 0.1, *  0.23, **  0.11, n.s  0.15, *** |
|  |  |  |  |
| **Season 3 (summer)** | | | |
| Within-tree asynchrony – Pollinators  Within-tree asynchrony – Parasites  Within-tree asynchrony – Seeds | -0.27, ***  -0.12, **  N/A | N/A  -0.073, ***  N/A | -0.27, *** -0.19, ***  N/A |
| Pollinators – Parasites  Pollinators – Seeds | 0.27, ***  N/A | N/A  N/A | 0.27, ***  N/A |
| Parasites – Seeds | N/A | N/A | N/A |
| SD of volume – Within-tree asynchrony | 0.34, *** | N/A | 0.34, *** |
| Volume – Pollinators  Volume – Parasites  Volume – Seeds  Volume – SD of Volume | 0.66, ***  0.18, **  0.53, ***  0.4, *** | -0.04, ***  0.15, ***  N/A  N/A | 0.63, ***  0.33, ***  0.53, ***  0.4, *** |
|  | | | |
| **Season 4 (wet)** | | | |
| **Relationship between** | **Direct effects** | **Indirect effects** | **Total effects** |
| Within-tree asynchrony – Pollinators  Within-tree asynchrony – Parasites  Within-tree asynchrony – Seeds | -0.06, n.s  -0.12, *  N/A | N/A  -0.011, n.s  0.019, n.s | -0.06, n.s  -0.128, **  0.019, n.s |
| Pollinators – Parasites  Pollinators – Seeds | 0.17, **  0.16, ** | N/A  -0.038, * | 0.17, **  0.122, * |
| Parasites – Seeds | -0.23, *** | N/A | -0.23, *** |
| SD of volume – Within-tree asynchrony | 0.66, *** | N/A | 0.66, *** |
| Volume – Pollinators  Volume – Parasites  Volume – Seeds  Volume – SD of Volume | 0.5, ***  0.32, ***  0.37, ***  0.25, *** | -0.01, n.s  0.06, *  -0.01, n.s  N/A | 0.5, ***  0.38, ***  0.37, ***  0.25, *** |
