# Supplementary material for: High Temperatures Result in Smaller Nurseries which Lower Reproduction of Pollinators and Parasites in a Brood Site Pollination Mutualism
Source: PLoS One. 2014 Dec 18;9(12):e115118. doi: 10.1371/journal.pone.0115118 (PMC4270730; doi:10.1371/journal.pone.0115118)
Supplement: S5 Table — Magnitudes of unexplained variance (U) for each factor in each of the best fit models for seasons 1 to 4. Magnitudes are represented as standardised path coefficients that range between −1 and +1. *** p<0.001, ** p<0.01 and >0.001, *p<0.05 and >0.01, n.s. p>0.5. (DOC) [file pone.0115118.s010.doc]

**Table S5. Magnitudes of unexplained variance (U) for each factor in each of the best fit models for seasons 1 to 4.**

Magnitudes are represented as standardised path coefficients that range between -1 and +1. *** p < 0.001, ** p < 0.01 and > 0.001, *p <0.05 and > 0.01, n.s. p > 0.5.

| **Season** | **Model no.** | **Factor** | **U** |
| --- | --- | --- | --- |
| 1 | 6b | Within-tree asynchrony  Pollinators  Seeds  Parasites  SD of parasites | 0.98, ***  0.77, ***  0.96, ***  0.85, ***  0.68, *** |
| 2 | 7b | Within-tree asynchrony  Pollinators  Seeds  Parasites  SD of parasites | 0.99, ***  0.72, ***  0.84, ***  0.99, ***  0.96, *** |
| 3 | 7a | Within-tree asynchrony  Pollinators  Seeds  Parasites  SD of volume | 0.94, ***  0.73, ***  0.85, ***  0.9, ***  0.92, *** |
| 4 | 1a | Within-tree asynchrony  Pollinators  Seeds  Parasites  SD of volume | 0.75, ***  0.87, ***  0.9, ***  0.9, ***  0.97, *** |
