# Supplementary material for: High Temperatures Result in Smaller Nurseries which Lower Reproduction of Pollinators and Parasites in a Brood Site Pollination Mutualism
Source: PLoS One. 2014 Dec 18;9(12):e115118. doi: 10.1371/journal.pone.0115118 (PMC4270730; doi:10.1371/journal.pone.0115118)
Supplement: S6 Table — Details of binomial GLMM analysis to examine effect of season on the proportions of non-pollinating parasitic gallers per syconium. Tree identity was used as a random factor in this analysis. The generalised linear mixed model (GLMM) was carried out using a logit link function (a binomial GLMM) with the data. (DOC) [file pone.0115118.s011.doc]

**Table S6. Details of binomial GLMM analysis to examine effect of season on the proportions of non-pollinating parasitic gallers per syconium.** Tree identity was used as a random factor in this analysis. The generalised linear mixed model (GLMM) was carried out using a logit link function (a binomial GLMM) with the data.

| Linear mixed-effects model fit by maximum likelihood | | | | | | |  |
| --- | --- | --- | --- | --- | --- | --- | --- |
| Data: dat | |  |  |  |  |  |  |
| AIC BIC logLik | | |  |  |  |  |  |
| NA NA NA | | |  |  |  |  |  |
|  |  |  |  |  |  |  |  |
| Random effects: | |  |  |  |  |  |  |
| Formula: ~1 | Tree | | |  |  |  |  |  |
| (Intercept) Residual | | | |  |  |  |  |
| StdDev: 0.2643804 0.3389038 | | | |  |  |  |  |
|  |  |  |  |  |  |  |  |
| Variance function: | | |  |  |  |  |  |
| Structure: fixed weights | | | |  |  |  |  |
| Formula: ~invwt | | |  |  |  |  |  |
| Fixed effects: gall_prop2 ~ season | | | | |  |  |  |
| Value Std.Error DF t-value p-value | | | | | | |  |
| (Intercept) -2.2405630 0.1423054 398 -15.744755 0e+00 | | | | | | |  |
| season2 -1.2698643 0.2462227 398 -5.157381 0e+00 | | | | | | |  |
| season3 -1.2893310 0.2278159 398 -5.659530 0e+00 | | | | | | |  |
| season4 -0.8656745 0.2130450 398 -4.063340 1e-04 | | | | | | |  |
| Correlation: | |  |  |  |  |  |  |
| (Intr) seasn2 seasn3 | | | |  |  |  |  |
| season2 -0.417 | | | |  |  |  |  |
| season3 -0.463 0.250 | | | |  |  |  |  |
| season4 -0.490 0.281 0.288 | | | |  |  |  |  |
|  |  |  |  |  |  |  |  |
| Standardized Within-Group Residuals: | | | | |  |  |  |
| Min Q1 Med Q3 Max | | | | | | | |
| -1.02939466 -0.44824687 -0.25131986 0.08360174 8.71792123 | | | | | | | |
|  |  |  |  |  |  |  |  |
| Number of Observations: 417 | | | |  |  |  |  |
| Number of Groups: 16 | | |  |  |  |  |  |
