# Supplementary material for: High Temperatures Result in Smaller Nurseries which Lower Reproduction of Pollinators and Parasites in a Brood Site Pollination Mutualism
Source: PLoS One. 2014 Dec 18;9(12):e115118. doi: 10.1371/journal.pone.0115118 (PMC4270730; doi:10.1371/journal.pone.0115118)
Supplement: S1 Text — Defining seasons based on environmental conditions. (DOC) [file pone.0115118.s012.doc]

**Supporting Information Text S1. Defining seasons based on environmental conditions**

We obtained data on the maximum and minimum daily temperatures, relative humidity (RH) and rainfall for each of the reproductive cycles per tree recorded (between Nov 2008 to Aug 2010) from the Centre for Atmospheric and Oceanic Sciences, Indian Institute of Science, Bangalore, India. All statistical analyses were conducted with the software R version 2.15.2. A principal components analysis (PCA) was conducted using the package *FactomineR* with the data on maximum and minimum daily temperatures and RH to define seasons based on these abiotic environmental factors. In order to segregate reproductive episodes according to common abiotic environmental conditions experienced by them, we used a clustering algorithm *cluster* to assign each reproductive episode per tree to each of the seasons obtained through the PCA.

The PCA analysis based on maximum and minimum daily temperatures and RH indicated the presence of four distinct clusters (Fig. S1) defining four seasons in a year between the years 2008 and 2010. These were season 1 (winter), season 2 (hot days and cold nights), season 3 (summer) and season 4 (wet). Season 1, or the winter season was defined by low temperatures (average high temperatures = 26°C ± 0.5°C, average low temperatures = 17°C ± 1°C) with RH ranging from 96% ± 2% (average high) to 48% ± 8% (average low) and lasted from November to January. Season 2, had hot days and cold nights with average high temperatures = 31°C ± 1°C and average low temperatures = 18°C ± 1°C; had the lowest RH ranges amongst the four seasons (average high RH = 84% ± 3%, average low = 24% ± 4%) and lasted through February and March. Season 3, was the hot season, being defined by high temperatures (average high temperatures = 32°C ± 1°C, average low temperatures = 21°C ± 0.3°C) with the second lowest RH values ranging from 90% ± 3% (average high) to 36% ± 6% (average low) and lasted from April to May. Season 4, was defined as the wet season with average high temperatures = 27°C ± 0.4°C and average low temperatures = 20°C ± 0.4°C; had the highest RH ranges amongst the four seasons (average high RH = 97% ± 1%, average low = 60% ± 2%) and lasted from June to October. Season 4 received the highest rainfall. Season 1 received occasional showers in December and season 3 received evening showers for 1 week in mid-April. Cluster analysis indicated that each reproductive cycle observed could be classified as belonging to one of these four defined seasons. Of the 94 total reproductive episodes (across 16 trees) recorded over the 20 months of observation, 16 were assigned to season 1, 22 to season 2, 26 to season 3, and 30 to season 4.

**Figure S1. MDS plot obtained from PCA analysis.**

MDS plot of the 94 reproductive episodes of *F. racemosa* trees, each of which was defined by environmental variables consisting of temperature and RH values across the duration of that reproductive episode. The different seasons in which these episodes occurred were season 1 (closed squares, ■), season 2 (open circles, ○), season 3 (open squares, □) and season 4 (closed circles, ●). The ellipses define the 95% confidence interval limit for each group around a barycentre calculated from the various points within that group.
