# Supplementary material for: High Temperatures Result in Smaller Nurseries which Lower Reproduction of Pollinators and Parasites in a Brood Site Pollination Mutualism
Source: PLoS One. 2014 Dec 18;9(12):e115118. doi: 10.1371/journal.pone.0115118 (PMC4270730; doi:10.1371/journal.pone.0115118)
Supplement: S2 Text — Details of path model construction and selection. (DOC) [file pone.0115118.s013.doc]

**Supporting Information Text S2. Details of path model construction and selection.**

Details of path model construction for *a priori* and alternative models, and the methodology followed to obtain best-fit and most parsimonious models.

We began by constructing an *a priori* model (Fig. 2, main text) based on known biological facts about the system to investigate the circular effects of within-tree asynchrony on various syconium inhabitants. Alternative models were constructed by removing the effect of various factors on others (detailed in Table S1) and compared with the *a priori* model to obtain the best-fit (using measures of model fit such as χ^2^ value, p-value and root mean square error of approximation (RMSEA)) and most parsimonious model (denoted by the degrees of freedom, which is equivalent to the number of unspecified paths in the model). The best-fit model thus obtained was further used to construct a two alternative models, one in which intra-crop variation in syconium volumes and the other in which intra-crop variation in the number of non-pollinators per syconium was expected to affect within-tree asynchrony (represented by standard deviation or SD of volume and SD of parasites per syconium in Fig. 2, main text). The second set of models were compared amongst themselves using the same measures of model fit and parsimony as described above to obtain a final circular model explaining the effects of within-tree asynchrony on syconium inhabitants with a feed-back cycle further affecting within-tree asynchrony (Table S1). This methodology was followed to obtain the best-fit and most parsimonious models for each of the 4 seasons.

In the table S1, pollinators (*C. fusciceps*), non-pollinators (*A. testacea* + *A. fusca* + *Apocrypta westwoodi* + *Apocrypta* sp. 2 + *A. agraensis*) and seeds refer to various syconium contents/inhabitants. The inter-syconium variation in syconium inhabitants were described using the SD in numbers of that inhabitant amongst syconia within a tree during a particular reproductive episode. Acceptable models are expected to have RMSEA (root mean square error of approximation) values < 0.06 and p-values ≥0.05. The model with maximum explanatory power has the highest p-value, low χ^2^ and low RMSEA values among all the models being compared. Model parsimony, which is measured by the number of unspecified/unestimated paths is indicated by the model’s degrees of freedom (df). The *a priori* model has the maximum number of estimated paths and the lowest df, whereas the most parsimonious model has the fewest estimated paths and the highest df.

For season 1, model 1 was found to be the best fit model in the first round of model selection. This was further modified into models 1a and 1b, which were compared. Model 1b was found to be the best fit model in this comparison and was therefore chosen as the final model for season 1 (winter).

For season 2, model 7 was found to be the best fit and most parsimonious model in the first round of model selection. This was further modified into models 7a and 7b, which were compared. Model 7b was found to be the best fit model in this comparison and was therefore chosen as the final model for season 2 (hot days and cold nights).

For season 3, model 7 was found to be the best fit and most parsimonious model in the first round of model selection. This was further modified into models 7a and 7b, which were compared. Model 7a was found to be the best fit and most parsimonious model in this comparison and was therefore chosen as the final model for season 3 (summer).

For season 4, model 1 was found to be the best fit model in the first round of model selection. This was further modified into models 1a and 1b, which were compared. Model 1a was found to be the best fit and most parsimonious model in this comparison and was therefore chosen as the final model for season 4 (wet).
