# Supplementary material for: High Temperatures Result in Smaller Nurseries which Lower Reproduction of Pollinators and Parasites in a Brood Site Pollination Mutualism
Source: PLoS One. 2014 Dec 18;9(12):e115118. doi: 10.1371/journal.pone.0115118 (PMC4270730; doi:10.1371/journal.pone.0115118)
Supplement: S3 Text — Relationship between external syconium volume and volume of lumen in B-phase syconia. (DOC) [file pone.0115118.s014.doc]

**Supporting Information Text S3.** **Relationship between external syconium volume and volume of lumen in B-phase syconia:**

A total of 30 B-phase syconia were collected from various trees and measurements of external volume and lumen volume carried out within an hour after collection. Using a vernier caliper, we first measured external ostiole–stalk length (EOS) and external syconium diameter (ED) (Figure S3, (i)), after which the syconium was sliced into two equal halves from ostiole to stalk in the same plane that was used to measure external syconium diameter. Measurements for the lumen involved one along the ostiole-stalk axis (IOS), and a second one to measure lumen diameter or internal syconium diameter (ID) at 90° to the ostiole–stalk axis (Fig. S6a, (ii)). These values were used in a standard formula for obtaining volumes of ellipsoids to obtain external syconium volume and lumen volume. A Pearson correlation test used to investigate the relationship between external syconium volume and lumen volume revealed a significantly positive relationship (Figure S4) with a correlation coefficient of 0.89 (t = 10.46, df = 28, p<0.0001).

**Figure S3. Measurements for external syconium volume and lumen volume.**

(i) uncut syconium, where EOS = External Ostiole–Stalk length, ED = External Diameter;

(ii) syconium cut to expose lumen, where IOS = Internal Ostiole–Stalk length, ID = Internal Diameter

**Figure S4. Positive correlation between external syconium volume and lumen volume.** Pearson correlation coefficient = 0.89, (t = 10.46, df = 28, p<0.0001).
