# Supplementary material for: High Temperatures Result in Smaller Nurseries which Lower Reproduction of Pollinators and Parasites in a Brood Site Pollination Mutualism
Source: PLoS One. 2014 Dec 18;9(12):e115118. doi: 10.1371/journal.pone.0115118 (PMC4270730; doi:10.1371/journal.pone.0115118)
Supplement: S4 Text — Variation in proportions of non-pollinating parasitic gallers per syconium across the four seasons. (DOC) [file pone.0115118.s015.doc]

**Supporting Information Text S4. Variation in proportions of non-pollinating parasitic gallers per syconium across the four seasons.**

To investigate if syconia collected across the four seasons differed in the number of gallers parasitizing them, we used a subset of the syconia collected for this study. Roughly 100 syconia per season were randomly chosen and the non-pollinating fig wasps in them (both males and females) were segregated according to whether they were gallers or parasitoids. We used this information to calculate proportions of gallers per syconium (number of gallers in a syconium/total number of syconium occupants which included numbers of pollinators, seeds and all non-pollinators). We then carried out a generalised linear mixed model (GLMM) using a logit link function (a binomial GLMM) with the data, the results of which indicated that seasons 1 and 4 had the highest proportions of galler progeny per syconium, and that the proportions of galler progeny per syconium in these two seasons were significantly different from those in seasons 2 and 3 (Figure S5 and Table S6).

**Figure S5. Proportions of gallers per syconium across the different seasons.**

The different letters above boxes represent significant differences at the p < 0.05 level (values with the same letters were not significantly different) as according to binomial GLMM analyses using tree identity as a random factor.
